# An ecological niche shift for Neanderthal populations in Western Europe 70,000 years ago

**DOI:** 10.1038/s41598-021-84805-6

**Published:** 2021-03-05

**Authors:** William E. Banks, Marie-Hélène Moncel, Jean-Paul Raynal, Marlon E. Cobos, Daniel Romero-Alvarez, Marie-Noëlle Woillez, Jean-Philippe Faivre, Brad Gravina, Francesco d’Errico, Jean-Luc Locht, Frédéric Santos

**Affiliations:** 1 University of Bordeaux, CNRS, MCC, PACEA, UMR 5199, Bâtiment B2, Allée Geoffroy St. Hilaire, CS 50023, 33600 Pessac, France; 2 Biodiversity Institute, University of Kansas, 1345 Jayhawk Blvd, Lawrence, KS 66045 USA; 3 CNRS, Département Hommes et Environnement, Muséum National d’Histoire Naturelle, Institut de Paléontologie Humaine, UMR 7194, 1 rue René Penhard, 75013 Paris, France; 4 Agence Française de Développement, 5 rue Roland Barthes, 75012 Paris, France; 5 Centre for Early Sapiens Behaviour, University of Bergen, Øysteinsgate 3 Post box 7805, 5020 Bergen, Norway; 6 Institut National de Recherches Archéologiques Préventives (INRAP) Nord-Picardie, 32 avenue de l’Etoile du Sud, 80440 Glisy, France; 7 CNRS, University of Paris 1, University of Paris Est Créteil, LGP, UMR 8591, 1 Place A. Briand, 92195 Meudon Cedex, France

**Keywords:** Ecological modelling, Archaeology

## Abstract

Middle Paleolithic Neanderthal populations occupied Eurasia for at least 250,000 years prior to the arrival of anatomically modern humans. While a considerable body of archaeological research has focused on Neanderthal material culture and subsistence strategies, little attention has been paid to the relationship between regionally specific cultural trajectories and their associated existing fundamental ecological niches, nor to how the latter varied across periods of climatic variability. We examine the Middle Paleolithic archaeological record of a naturally constrained region of Western Europe between 82,000 and 60,000 years ago using ecological niche modeling methods. Evaluations of ecological niche estimations, in both geographic and environmental dimensions, indicate that 70,000 years ago the range of suitable habitats exploited by these Neanderthal populations contracted and shifted. These ecological niche dynamics are the result of groups continuing to occupy habitual territories that were characterized by new environmental conditions during Marine Isotope Stage 4. The development of original cultural adaptations permitted this territorial stability.

## Introduction

One of the major goals of archaeology is to evaluate the factors that influenced past cultural adaptations and, in turn, identify the mechanisms behind culture-environment relationships^1^. Human populations have the potential to respond rapidly to climatic and environmental change via culturally mediated behaviors (e.g., technology, subsistence, settlement strategies) developed within cumulative cultural systems that can evolve over the course of centuries or even decades^2^. This potential can be expressed in a variety of forms and at varying rhythms. It has been argued, for example, that the Late Glacial recolonization of Northern Europe was not continuous but proceeded in pulses^3,4^. The extent to which past human populations of the Middle and Upper Paleolithic of Europe and the Middle and Later Stone Ages in Africa responded to climate and environmental change likely varied through time and between regions. Recent research has demonstrated that Neanderthals relied on cumulative culture and that such behavior was not restricted solely to Anatomically Modern Humans^5–7^. However, little focus has been paid to Neanderthal behaviors within the framework of the ecological niches they exploited, nor how these niches and cultural adaptations may have evolved in response to pronounced environmental change. Some have estimated population distributions without focusing on associations between techno-typological systems and their diachronic variability^8,9^. Others have studied Neanderthal technological and subsistence variability through time^10–12^ and framed interpretations against inferred demography, settlement systems, or climatic variability without quantitatively taking into account ecological niches. Thus, it remains unclear how culture-ecology relationships within particular Neanderthal regional trajectories varied through time.

Morphological and genetic data indicate that the Neanderthal clade emerged after 700 thousand years ago (ka), and mosaics of Neanderthal morphological features appear in the European fossil record ca. 450 ka during Marine Isotope Stage (MIS) 12^13,14^. Archaeological assemblages dated to MIS 11–10 include technological innovations developed by populations within the *H. neanderthalensis* lineage^15^, and advanced flake-based industries (e.g., the Levallois technological system) emerge by at least MIS 8 (ca. 300 ka). Hominins unequivocally recognizable as morphologically Neanderthal were present in Europe by MIS 7, ~ 200 ka^13^, although it is still unclear how these populations were structured across the European landscape. Rogers and colleagues^14^ conclude that, while groups remained small, this was not necessarily the case for the overall population. Other genetic analyses^16,17^ suggest that Neanderthal groups were small and isolated, a hypothesis supported by low heterozygosity^18^. These findings indicate that the Neanderthal archaeological record reflects the activities of relatively small and regionally constrained populations—a pattern supported by isotopic evidence and patterns of lithic raw material transport^19^.

Here we employ ecological niche modeling methods^20^ to estimate existing fundamental niches of Middle Paleolithic Neanderthal populations within a geographically constrained area and across a period marked by pronounced changes in lithic technology, as well as significant climatic change, subsequent environmental reorganization, and changes in resource variability and predictability associated with an Interglacial–Glacial transition—the terminal phase of MIS 5 (MIS 5a: ca. 82–70 ka) and MIS 4 (ca. 70–60 ka). This period is associated with a sufficient number of chronologically attributable sites such that robust ecological niche modeling and archaeological assemblage analyses can be conducted effectively and produce reliable results. This situation makes the targeted chronological interval an excellent candidate for examining Neanderthal culture-ecology relationships.

In light of inferred Neanderthal population structure, we focus on the archaeological record of a large area of Western Europe geographically constrained by the Pyrenees to the south, the Alps and Jura Mountains to the east, the Atlantic Ocean to the west, and the northern limit of observed sites. While terrestrial barriers would not have prevented movement between the study area and neighboring regions, genetic and archaeological evidence suggests that they did play a role in the definition of regional Neanderthal territories^17–19,21,22^, thus rendering our targeted region archaeologically pertinent. Archaeologists have conducted site investigations and surveys in the study area for well over a century, and we assume, especially considering the intensity of archaeological fieldwork paired with the large-scale, systematic surveys performed over the last few decades, that the samples of archaeological sites attributed to our periods of interest are representative. This same argument has been advanced for examinations of the Upper Paleolithic record^23^, and it is equally applicable to the Middle Paleolithic record since archaeological and geoarchaeological surveys typically document all observed sites and not just those for a particular time period to the exclusion of others.

The archaeological record for MIS 5a and MIS 4 is dominated by stone tool (lithic) production systems and lithic tools, and the composition of regional and temporally coherent series of lithic assemblages is commonly used to infer past cultural taxonomic units. It is argued^24–26^ that definitions of Neanderthal archaeological cultural entities should rely on lithic production systems that can be exclusive or co-occur with one another within individual archaeological assemblages. In the region targeted by this study and the population of sites chronologically attributed to the targeted time intervals (Supplementary Table S1), MIS 5a is dominated by the Levallois concept (Tables S2, S3), which relied on particular core reduction sequences to produce highly predetermined, standardized flake blanks^27^. This technological system is also common during MIS 4 (Tables S4, S5). A number of sites are characterized by a lithic technocomplex (LTC) composed only of Levallois methods of blank production, while numerous others reveal LTCs in which additional systems, such as those that produce blades or bifaces, were employed in conjunction with Levallois production methods. The Discoid reduction system is also present in both MIS 5a and MIS 4. While it is commonly associated with Levallois technology, in some assemblages it is exclusive. Discoid technology relied on cores—possessing one or more unprepared flaking surfaces—whose reduction continuously maintained suitable flaking angles between non-hierarchized flaking surfaces^28^. This technological system was geared around the production of identical or highly morphologically similar flake blanks, and it thus shares some similarities with Levallois systems. The Quina LTC^29–31^ appears during MIS 4 as a geographically and chronologically coherent technical system. This highly original system is built around the production of large, thick, asymmetrical blanks (flakes) with at least one elongated edge opposite a thick face^25,29^, and the use of scalariform retouch to transform blanks into a variety of tool forms. Blanks and retouched tools could be transformed when needed into cores for the production of smaller blanks with similar morphologies to be used unretouched or made into retouched tools^25^. The resulting assemblages typically have high frequencies of thick, convex scrapers with stepped, scalariform (Quina) retouch and few to no elements produced via Levallois production methods^30^. The Quina system represents a sharp contrast to Levallois systems since with the latter initial tool form was pre-conceived during blank production. The archaeological record of the study region depicts regionally and temporally varying frequencies of LTCs and associated technological products.

The principal utility of applying ecological niche modeling methods to the archaeological record is that they allow human behavioral phenomena to be placed in an ecological context. In so doing, it is possible to determine whether changes in cultural adaptions are associated with a conservation, expansion, or contraction of an archaeological population’s ecological niche through time, including intervals characterized by environmental change. Additionally, such methods make it possible to evaluate to what degree changes in material culture patterning were influenced by cultural and ecological factors. Such an approach can play an important role in understanding and interpreting culture-environment relationships through time—relationships that are difficult, if not impossible, to detect via examinations of environmental proxies and archaeological assemblages from isolated, geographically dispersed locations alone (i.e., archaeological sites).

With respect to correlative ecological niche modeling applied to archaeological data, predictive architectures allow the ecological niche occupied by an archaeologically defined cultural taxonomic unit (i.e., archaeological “culture”) during a given period to be estimated in both environmental and geographic dimensions via the geographic locations of archaeological sites where its characteristic material culture remains are recognized, as well as chronologically pertinent paleoenvironmental data. Predictive algorithms use these data to identify the environmental parameters shared among the archaeological sites and define the relationships between these parameters. An important capacity of these modeling architectures is that they permit the examination of niches between periods such that it becomes possible to determine whether successive technocomplexes exploited different niches. As it has been demonstrated that population size does not govern hunter-gatherer cultural complexity^32^, when comparing the technologies of successive archaeological populations and taking into account the environmental frameworks within which they operated it is possible to evaluate whether changes in material culture reflect the influence of ecological factors.

We use the Biotic-Abiotic-Mobility (*BAM*) framework^20^ to describe factors that constrain a population’s ecological niche and its geographic distribution. Following the Eltonian noise hypothesis^33^ and considering our geographic scale and the spatial resolution of our employed data layers, we assume that biotic interactions did not have a limiting effect on Neanderthal distributions. Since our models need to be transferred to distinct scenarios, such an assumption allows projections to be performed with fewer uncertainties. Therefore, our niche estimations correspond to the intersection between suitable environmental conditions (*A*) and accessible area (*M*)—an intersection representing the existing fundamental niche of Neanderthal populations that occupied the study region during two successive climatic intervals (i.e., MIS 5a and MIS 4). To estimate, evaluate, and compare Neanderthal niches for these two marine isotope stages, we employ the kuenm^34^ and ellipsenm^35^ R packages (see “Methods”). To provision the different correlative predictive methods, we use the geographic coordinates of archaeological sites within the study region that can be reliably attributed to either MIS 5a or MIS 4, along with climatic and vegetation variables derived from two paleoclimatic simulations primarily constrained by orbital parameters and that correspond to 80 and 60 ka (see “Methods”). These latter data are employed as proxies representing environmental conditions during MIS 5a and MIS 4, respectively. In our region of study, LTCs that incorporate the Levallois concept dominate MIS 5a, and this concept is replaced by the Quina system in certain areas during MIS 4. The ecological frameworks behind the geographic and temporal variability of these lithic technologies remain unexplored.

## Results

### Ecological niche estimations

**Figure 1:**
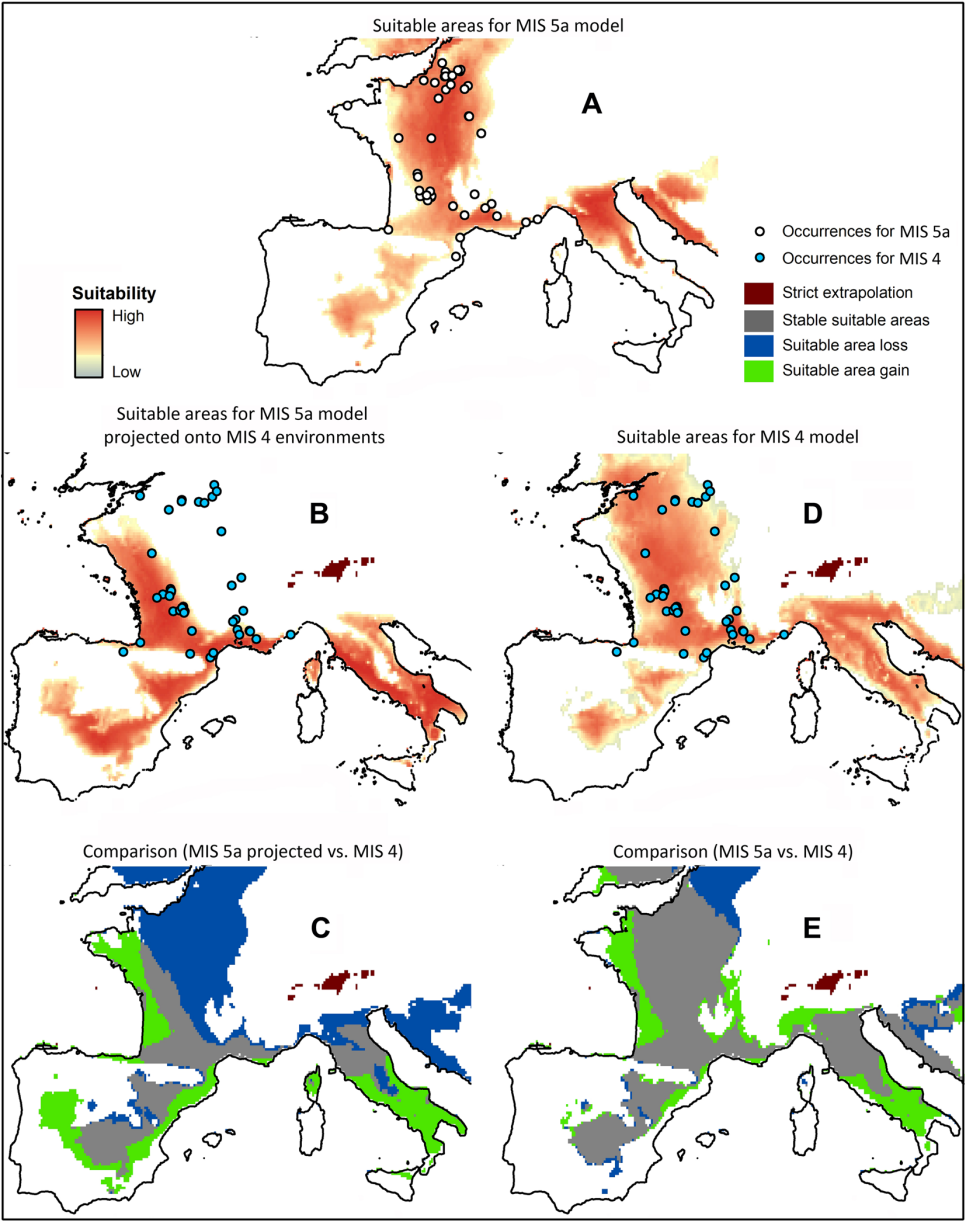
Geographic projections of Neanderthal ecological niche estimations produced with the kuenm R package. (**A**) Reconstructed Neanderthal niche for MIS 5a with coastlines reflecting 80 ka sea level (− 20 m)^36^; (**B**) Neanderthal MIS 5a niche projected onto MIS 4 environmental conditions with coastlines reflecting 60 ka sea level (− 60 m)^36^; (**C**) Comparative overlap of predicted suitable areas between the MIS 5a Neanderthal niche and the same MIS 5a niche projected onto MIS 4 environmental conditions; (**D**) Reconstructed Neanderthal niche for MIS 4; E) Comparative overlap of predicted suitable areas between the MIS 5a Neanderthal niche and the MIS 4 niche. Maps were created using ArcGIS 10.5.1 (https://desktop.arcgis.com/en/).

The geographic projection of the MIS 5a Neanderthal ecological niche exhibits high suitability scores along the Mediterranean coast, along the western margins of the Massif Central, up through present-day central France, the Paris Basin, and areas immediately to the north (Fig. 1A). High suitability scores for this niche are also expressed in northern Italy and the eastern coast of the Adriatic. Medium to medium–low suitability scores are present in central and northeastern portions of the Iberian Peninsula. It is important to point out that areas of suitable habitat present outside of the study area are those that share ecological similarities with the regions in which occurrence data are present but were not necessarily occupied by the same groups.

When the MIS 5a niche is projected onto MIS 4 glacial conditions, its suitable areas are compressed to the south and southwest, and this temporally projected niche fails to predict roughly one-third of the archaeological sites present during the latter period (Fig. 1B). This compression and southerly geographic displacement of MIS 5a suitable areas during MIS 4 is clearly illustrated when binary versions of the projected MIS 5a and MIS 4 niche predictions are superimposed (Fig. 1C). The northern and northeastern portions of the study region are characterized by a loss of suitable areas, while gains in suitable area are observed along the extreme western margins of the study area.

The MIS 4 ecological niche prediction is more geographically dispersed than that of the preceding period and is generally characterized by slightly lower suitability scores. During this period, the Neanderthal niche occupies largely the same regions as observed during MIS 5a, albeit with a loss of territories in the extreme north, and moderate extensions into the present-day Nouvelle Aquitaine and Bretagne regions, as well as southern regions of the Italian Peninsula (Fig. 1D). These patterns are readily visible when binary predictions for the two periods are superimposed (Fig. 1E).

**Figure 2:**
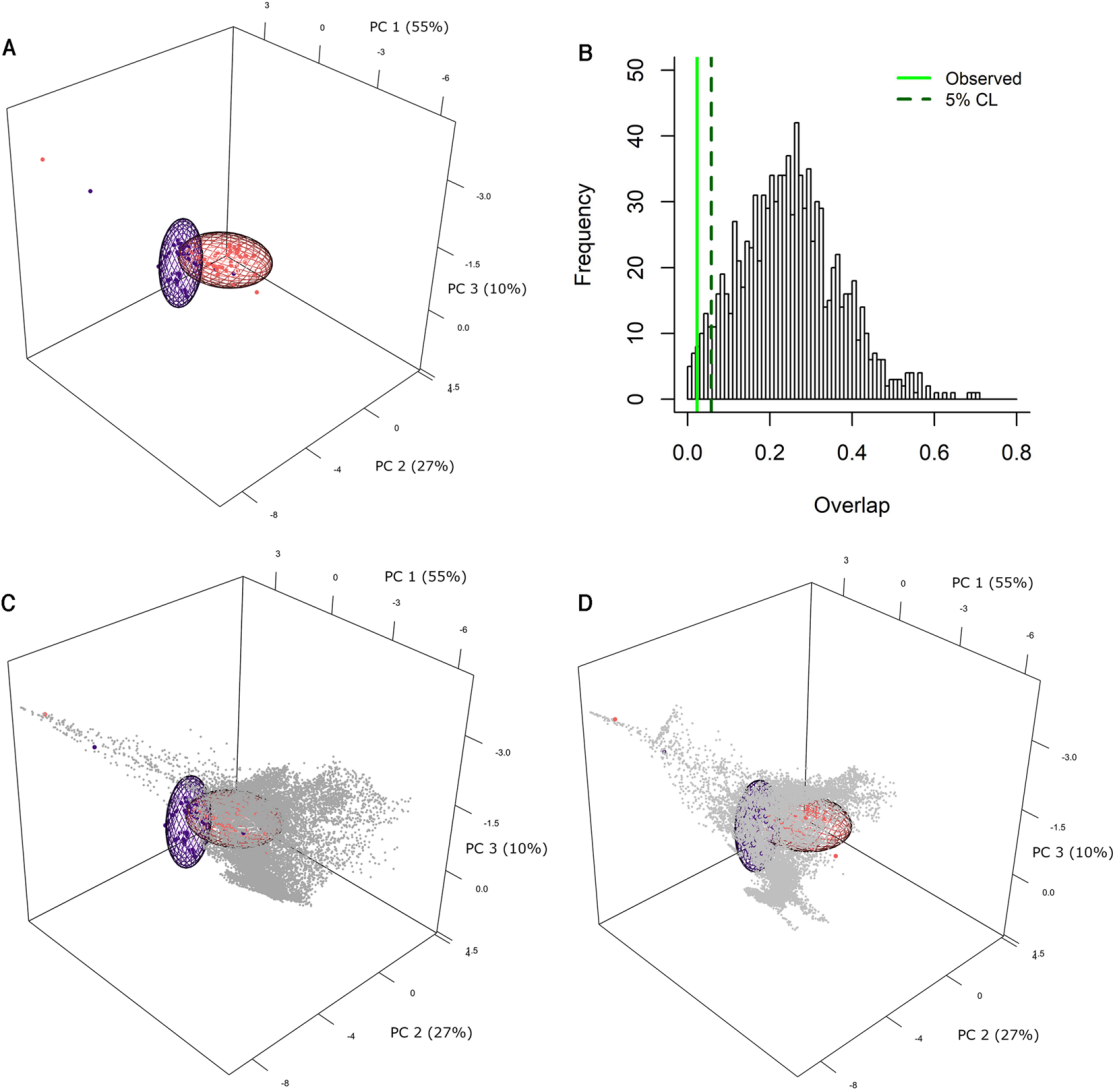
Minimum volume ellipsoid (MVE) niche estimations for MIS 5a and MIS 4 Neanderthals and their overlap. MIS 5a occurrences and ellipsoid: red; MIS 5a environmental background: dark grey; MIS 4 occurrences and ellipsoid: blue; MIS 4 environmental background: light grey. Note that MIS 5a has two occurrence points (archaeological sites) and MIS 4 has a single occurrence point that fall outside the reconstructed ellipsoid. These outlying occurrence points fall below the error parameter *E* set at 5%^37^.

The pattern of suitable area stability, loss, and gain reflected in the geographic projections of the niche estimations are replicated in the comparison of the MIS 5a and MIS 4 niche reconstructions performed in purely environmental dimensions. Minimum volume ellipsoid (MVE) estimations (Fig. 2; Figs. S1–S3) show that the two niches slightly overlap with one another, yet this small environmental overlap has a large geographic expression (Fig. 1E). The MVE reconstructions demonstrate that between MIS 5a and MIS 4 the Neanderthal niche underwent an important contraction and shift (Table S6). Each environmental niche envelope occupies conditions not present in the other, and these differences are significantly greater than one would expect to occur by chance, thus indicating that the shift in the Neanderthal niche between the two periods is significant (*p* = 0.014) (Fig. 2B). Ecological niche estimations in environmental dimensions produced with centroid and covariance matrix ellipsoids show the same pattern, but the observed niche shift is not statistically significant at the 95% threshold (*p* = 0.103; Table S7; Figs. S4–S7), although its *p*-value remains low. This difference derives from an optimization of the MVE algorithm that aims to reduce ellipsoid volume while maintaining the same proportion of points in the environmental envelope. Despite the *p*-values’ differing significance levels, both methods clearly demonstrate a shift in the ecological niche through time—a shift that is supported by the Maxent models and that would not be evident via an examination of occurrence distributions alone.

Examinations of ecological niche suitability scores between the northern and southern regions of the study area (see “Methods”) indicate that during MIS 5a the northern area is associated with slightly higher suitability scores than the southern area, the majority of each region’s scores fall within a relatively narrow range, and the overlap between regions corresponds to scores between 0.5 and 0.6 (Fig. S8). Additionally, four sites in the southern area are associated with the lowest suitability scores for this period’s estimated niche. The density plot of suitability scores for MIS 4 occurrence data illustrate a markedly different pattern than that of the preceding period (Fig. S9). A large portion of sites in the southern portion of the study region has suitability scores centered on 0.6, with a small number of sites possessing scores between 0.3 and 0.5. Inversely, scores for sites in the northern region of the study area are more variable and do not display a clear peak.

### Lithic technocomplex variability

Concerning lithic production systems and the suitability scores associated with the sites at which they are observed, the northern region during MIS 5a is dominated by LTCs composed of Levallois productions systems, often associated with laminar reduction sequences. These Levallois LTCs display elevated suitability scores (Fig. S10). In the south, LTCs based on Levallois production systems also dominate, but they are associated with slightly lower, and a broader range of, suitability scores. A Fisher’s exact test performed on the frequencies of the different LTCs between the northern and southern portions of the study area during MIS 5a demonstrates that the two regions differ significantly (Fig. S11).

During MIS 4, Levallois and Quina production systems predominate, and the latter is only present in the study area’s southern region. Examinations of regional differences between the suitability scores associated with these technological systems demonstrate that Levallois ecological niche suitability scores in the north are relatively low and range between 0.3 and 0.5, with a median value slightly below the latter (Fig. S12). In the southern region, LTCs that incorporate Levallois production have much higher suitability scores than those in the north, and those for the Quina are slightly higher and with a more restricted range (Fig. S12). LTC frequencies between the study area’s regions differ significantly during this latter chronological interval (Fig. S13). As would be expected, when LTC frequencies are considered for the entire study area, they differ significantly between MIS 5a and MIS4 (Fig. S14). This result is primarily due to the important presence of laminar production systems in the north during MIS 5a and the subsequent appearance of the Quina system in the south during MIS 4. This pattern is visible in Figs. S15 and S16, and Fisher’s exact evaluations of LTC frequencies for each specific region between MIS 5a and MIS 4 result in *p*-values that allow null hypotheses of no difference to be rejected. A Multiple Correspondence Analysis (MCA) of archaeological sites and their associated lithic production systems during the two chronological intervals confirms the differences described above between Quina and non-Quina systems (Fig. S17). Quina assemblages cluster separately from those in which Levallois methods are present and are associated with high suitability scores. Suitability score did not participate in the construction of the MCA axes, but plotting it in relation to the two axes (Fig. S17) shows that it is not strongly correlated with either and that Discoid assemblages have suitability scores that are slightly below the mean.

Fisher’s exact examination of the frequencies of Levallois methods recognized in site assemblages across the entire study region between MIS 5a and MIS 4 does not allow the null hypothesis of no difference to be rejected (Fig. S18). While this result is not significant, we view it with caution since there are a number of assemblages, especially for MIS 5a, that are labeled as “indeterminate” either due to a paucity of lithic remains or to the fact that no technological determination is available in the published literature. Due to the elevated number of “Indeterminate” classifications, more targeted examinations of Levallois method frequencies were not pursued.

Most Fisher’s exact tests performed on frequencies of lithic technology products recognized in archaeological levels of the employed sites produce *p*-values that allow null hypotheses of no difference to be rejected. These concern probability tests of observed frequencies for MIS 5a versus MIS 4 for the entire study area, as well as evaluations between north and south for each MIS and for the southern region between the two periods, and they are illustrated in Figs. S19–S21 and S22, respectively. As was observed with LTC variability within and between regions and the two Marine Isotope Stages, the observed significant differences are due primarily to variability in the frequencies of blades, bifaces, Quina blanks and centripetal flakes. It is noteworthy that in the northern portion of the study area, between MIS 5a and MIS 4, the null hypothesis of no difference cannot be rejected with respect to differences in the frequencies of lithic technology products, and this despite marked reductions in the frequency of most products during the latter period (Fig. S23).

## Discussion

Between MIS 5a and MIS 4, Neanderthal populations continued to occupy largely the same territories, with a concentration of sites in the Paris Basin and surrounding areas, as well as an important density of sites in southern regions of the study area. Based on recent syntheses of the character and distributions of archaeological assemblages^22,38–41^, it can be inferred that small and relatively isolated Neanderthal groups^14,16^ predominantly exploited well-defined regional territories. Across the Interglacial–Glacial transition, it is this territoriality that underpins the observed ecological niche shift. Rather than following the displaced geographic footprint of previously exploited environmental conditions across this period of climate change, Neanderthal populations instead continued to exploit largely similar territories subsequently characterized by different ecological conditions during MIS 4. This pattern of Neanderthal groups predominantly maintaining specific territories is also observed in the study area’s early Middle Paleolithic archaeological record in which technological methods are regionally differentiated, and it has been hypothesized that these differences prefigure later regionalization^42^.

Continued use of these same territories during MIS 4, many associated with markedly different environmental regimes, would have been facilitated by new cultural adaptations—an example being the Quina lithic technocomplex. A striking pattern of LTCs for which the Quina production system was a component is that the majority of these occurrences (excepting the sites of Vergisson and Chaumette in east-central France) are predominantly situated within a northwest to southeast trending distribution that skirts the northernmost areas of the projected MIS 5a niche prediction (projected onto MIS 4 environmental conditions) (Fig. 1B). A handful of Quina and debated Quina^43^ occurrences situated to the southeast and east in the Rhône and Saône River valleys, outside the principal Quina area, differ with respect to technological features and frequencies of certain classes of tools^43–45^ and may represent cultural diffusion of technical ideas to these regions, although the possibility of cultural convergence cannot be dismissed out of hand. The northwestern parts of the core region, as well as those to the west along the Atlantic coast, represent gained suitable area during MIS 4 and were dominated by cold, humid conditions and boreal forest during the MIS 5a-4 transition and the first half of MIS 4^46^. Thus, these Quina sites were associated with environmental conditions that characterized the most northern expressions of the Neanderthal niche during MIS 5a^47,48^. Additionally, within the MIS 4 ecological niche estimation, Quina occurrences are associated with elevated suitability scores relative to LTCs in which this production system is absent (Figs. S12, S17).

Certain early Middle Paleolithic (MIS 9–6) lithic assemblages from southern France feature structured and segmented core reduction sequences, as well as tool production strategies and retouched tools that share some similarities with the Quina system^42,49–52^. However, it is only during MIS 4 that Neanderthal groups in the southwestern portion of the study area develop the Quina system. We argue that such development in a geographically well-constrained region, occurring in conjunction with the dramatic shift in this region’s environmental conditions during MIS 4, is a product of certain groups drawing upon a deep cultural repertoire of technical knowledge to expand upon certain technical solutions in response to changes in resource predictability. An emergence from a local cultural substrate would better explain the coherent structure of the Quina system than would the hypothesis of intrusive Neanderthal groups bearing a new technological tradition.

Mobility^53^ and technological innovation^54^ are two manners in which hunter-gatherer populations can respond effectively to changes in resource predictability brought about by climate change and subsequent environmental reorganization. With its emphasis on retouched tool design, as well as core-on-flake reduction^25^, the Quina system was highly flexible^55^ and able to produce easily transportable, multifunctional tools that could be resharpened multiple times or transformed into cores or other tool forms over extended periods of time^56^. While the exploitation of lithic source areas by groups that employed the Quina system does not differ from the preceding period during which Levallois systems were predominant^41^, the flexibility of the Quina system would have allowed human groups to become less tethered to specific lithic raw material sources and employ different mobility and hunting strategies paired with this novel technology.

In the northern regions of the study area during MIS 4, numerous archaeological occurrences are situated along the northeastern margins of the estimated niche in areas characterized by moderate to low suitability measures (Figs. S12, S17), and their lithic assemblages are largely dominated by flakes produced via Levallois technology. This is in significant contrast to MIS 5a during which diverse Levallois and blade industries that occasionally incorporated bifacial tools (Fig. S15) are observed and associated with higher suitability scores (Fig. S10). This pattern is of interest because these regions would have been largely dominated by open, periglacial landscapes^47^ supporting steppe-tundra vegetation with occasional open woodland^48^ and populated by cold-adapted migratory game animals (i.e., reindeer)^38^—conditions in which ethnographically-documented hunter-gatherers favor highly reliable and maintainable toolkits^57^. This expectation differs from Levallois dominated LTCs focused on the production of flake blanks that are highly predetermined in form and commonly contain a large variety of tool forms^25^. One hypothesis to explain this pattern in lithic technology is that during MIS 4 Neanderthals in these northern regions restructured their settlement and mobility strategies so that raw material source areas were more frequently encountered. Such land-use patterns would have allowed the continued use of a less flexible technological system reliant on core reduction to determine tool form. In other words, we propose that Neanderthal behavioral innovation in these northern areas is principally reflected in the organization of movements across the landscape rather than stone tool production methods. Better documenting access to and circulation of lithic raw materials in regions north of the Loire River valley would prove key for evaluating this hypothesis.

Between MIS 5a and MIS 4, Levallois production systems continued to be predominant in northern areas of the study region, while in a large proportion of archaeological contexts south of the Loire we observe the appearance of the Quina technological system. This pattern, when paired with the fact that during MIS 4 differences in the frequency of lithic production systems between the northern and southern portions of the study area are significant (Fig S13), further supports the hypothesis that Neanderthal groups were organized into regional populations and that cultural interactions did not occur at a scale sufficient for transmitting and maintaining technological innovations between them. Perhaps most telling is the fact that the flexible Quina system and its readily maintainable tool forms are absent during MIS 4 in northern areas where it would have been well suited.

## Conclusion

Our approach demonstrates for the first time that between MIS 5a and MIS 4 the ecological niche of culturally cohesive Neanderthal populations in Western Europe contracted and shifted. Some of these populations elaborated highly adaptive cultural innovations in order to continue exploiting habitual territories whose environmental characteristics were affected by pronounced climate change. Continually exploiting the same territories across periods of dramatic environmental change requires cultural flexibility as previous cultural adaptations may no longer be effective in the face of new ecological conditions. Such flexibility is manifested in areas south of the Loire River Valley by the appearance of the Quina lithic production system during MIS 4. When systematically integrated into archaeological research, the approach employed here permits archaeologists to approach questions from a different angle and to make better-informed inferences with respect to cultural adaptation and cultural evolution. Our approach can play a key role in investigations of older periods in efforts to determine when humans began to employ cultural innovation as a strategy to respond to climatic challenges.

## Methods

### Occurrence data

We mined the published literature to obtain an initial corpus of sites in the study area that contained archaeological assemblages reliably attributed to Neanderthal occupations. Next, we eliminated those for which no chronological attribution was possible or for which such attributions were uncertain. Of the remaining sites, we eliminated those for which an unequivocal attribution is not possible to either Marine Isotope Stage (MIS) 5a or MIS 4 via chronological data or proxies. The relatively coarse resolution of dating methods for this period and imprecision associated with other chronological proxies do not allow one to attribute an archaeological site to a specific portion of a MIS (e.g. early MIS 5a versus terminal MIS 5a). Due to this constraint, we grouped archaeological sites by Marine Isotope Stage (MIS). There exist site levels associated with chronological measurements whose standard error interval place the level within the latter portion of MIS 5a and the early stages of MIS 4. In such instances, we examined other data (faunal spectra, sedimentological characteristics, etc.), if available, that could aid in determining whether a level in question could be associated with interglacial or glacial environmental conditions, and the appropriate temporal designation was made. In instances, where no other data were available, the site was excluded from the final occurrence dataset (Supplementary Table S1).

### Lithic technological data

We assembled and summarized lithic technological data associated with archaeological levels, chronologically attributed to either MIS 5a or MIS 4 (Tables S2 and S4, respectively), documented at the sites employed as occurrence data to estimate ecological niches. Because available lithic technological data derive from fieldwork conducted decades ago, as well as modern excavations, their quality is variable. For example, during the first half of the twentieth century, investigators sometimes only kept representative samples of excavated artifacts or, during the course of analysis and handling over the years, artifacts have been misplaced^58^. Furthermore, the excavated surface areas between sites vary, as does prehistoric site function. These factors make it difficult to draw robust conclusions from analyses of tool type frequencies, for example. Despite these limitations, it remains possible to conduct analyses pertaining to lithic production systems and the technical products that issue from these systems (e.g., Levallois flakes, Quina blanks, chordal flakes, etc.) since it is possible to determine technological strategies from non-exhaustive samples of lithic material culture remains. We conducted a variety of statistical analyses on such technological data using R 3.6.1^59^, along with the FactorMineR package^60^ for multiple correspondence analyses and the Lattice package^61^ for density plots. Tables S3, S5, and S8 present the presence or absence of the analyzed lithic technological variables in various formats depending on the type of conducted analysis. In order to examine technological variability within each Marine Isotope Stage, we also divided the study area into two broad regions labelled “North” and “South.” The distribution of archaeological sites and lithic production systems, along with the handful of regionally focused lithic raw material circulation studies^22,41,62^, indicate that the lower two-thirds of the Loire River serves as a boundary between these two regions. The division between north and south runs from the southern portion of the Pays de la Loire/the northern portion of Nouvelle-Aquitaine across the study area in a slightly southeasterly direction to the Auvergne-Rhône-Alpes region.

### Paleoclimatic simulations

Paleoclimatic simulation work employed the Atmosphere–Ocean General Circulation Model (AOGCM) IPSL CM5A (Institut Pierre Simon Laplace Climate Model version 5A)^63^ composed of the LMDZ atmosphere model coupled to the ocean OPA8/NEMO model via the OASIS coupler. For this analysis, we retained the simulations performed by M–N Woillez for 80 ka (MIS5a) and 60 ka (MIS4) as they are good proxies for the two Marine Isotope Stages that we target. Boundary conditions for these two simulations are: (a) present-day ice sheets for MIS 5a and ICE6-G interim reconstruction at 16 ka^64^ as an analogue for the MIS 4 ice sheets; (b) Greenhouse gases according to ice core records^65^ ; and (c) Orbital parameters^66^ (Table S9). Both simulations ran for approximately 600 years, at which point surface climatic variables reached equilibrium, and averages were calculated over the last 60 years of each simulation. To increase spatial resolution of the IPSL CM5A-generated simulations, we downscaled them to 0.16° via a generalized additive model^67^. Downscaled outputs were used to drive the process-based global vegetation model LPJ-LMfire^68^ and simulate vegetation in Europe off-line, meaning that there was no vegetation feedback on climate. We retained only net primary productivity (NPP) and biomass (total carbon in living biomass: g/m^2^), and these, along with the simulated climatic outputs of coldest month temperature, warmest month temperature, and mean annual precipitation, were used to estimate past ecological niches for Neanderthal populations during MIS 5a and MIS 4.

### Ecological niche modeling

We used ecological niche modeling methods to characterize Neanderthal niches for MIS5a and MIS4. Data preparation for modeling, model calibration, final models and transfers, post-modeling analyses, and assessment of extrapolation risks were performed in R 3.6.1^59^ using the kuenm R package^34^, which employs Maxent 3.4.1^69^ as the modeling algorithm. Inputs were the retained variables produced by the climatic and vegetation simulations along with the geographic coordinates of Neanderthal archaeological sites (Table S1) chronologically attributable to one of the targeted scenarios and situated within the defined study region.

We first performed model calibration by testing the performance of 2210 candidate models. We produced these models by using 26 distinct variable sets, which were made up of all unique combinations of more than two of the five climatic and vegetation variables described above^70^. Also employed were 17 regularization multipliers (0.1–1.0 at intervals of 0.1, 2–6 at intervals of 1, 8, and 10), along with five feature classes or feature class combinations (q, qp, lp, lq, lqp: l = linear, q = quadratic, p = product). All models were produced with a maximum of 10,000 random points as background. We evaluated model performance by first evaluating significance and predictive power via the partial ROC (created with 500 iterations, and 50 percent of data for bootstrapping)^37^ and omission rate metrics. This step employed 25% of randomly selected occurrence points against models created with the remaining 75% of occurrence points and allowing for a maximum error (*E*) of 5%^71^. We next evaluated model complexity via the Akaike Information Criterion (AIC) for small sample sizes (AIC_c_)^72^. We selected model parameterizations in the following order: those that resulted in statistically significant results, those with omission rates lower than the defined *E*, and those that had ΔAIC_c_ values lower than two. We used the selected parameters to create final models with 10 replicates by bootstrapping. Finally, we projected models for MIS5a onto MIS4 environmental conditions. During the process of model projection, we allowed free extrapolation given the response curves (i.e., response curves not truncated for at least two variables) observed during model calibration. In order to take into account the risks associated with strict extrapolation and to prevent misinterpretation of transferred areas with non-analogous conditions, we employed the mobility-oriented parity (MOP) metric^73^.

To evaluate how ecological niches differed between MIS5a and MIS4, we employed two approaches. We examined differences in the geographic distributions of suitable areas between the two periods via niche estimation and niche projection methods using the KUENM package. To conduct the comparisons, we calculated a median model based on the replicates produced with the retained, best-performing parameter settings and this for MIS5a, MIS5a projected onto MIS4, and MIS4. We thresholded each median model by reclassifying as non-suitable all grid cells with suitability scores within the bottom 5% of all values from grid cells containing an occurrence point, thereby creating a binary prediction (i.e., 0 = non-suitable; 1 = suitable). Comparisons between these binary predictions (MIS 5a vs. MIS5a projected onto MIS4; MIS5a vs. MIS4) allowed for the detection of suitable areas that remained stable between MIS5a and MIS4, as well as regions in which suitability was either lost or gained. Such comparisons also allow one to detect niche expansion, under assumptions of niche conservatism, via the identification of suitable areas during MIS4 that are predicted as non-suitable when projecting models from MIS5a.

To complement the kuenm analyses, we examined the degree of overlap in environmental dimensions between ellipsoid envelope ecological niches estimated using the ellipsenm R package^35^. We created ellipsoids, in a manner similar to Nuñez-Penichet et al.^74^, using environmental values associated with the archaeological occurrences for each period. We obtained environmental values by summarizing variables using a principal component analysis (PCA) performed with kuenm, and we retained the first three principal components (PCs) to measure overlap (Table S10). We first performed the PCA on the MIS 5a environmental variables. We then transformed (scaled and centered) the MIS 4 variables to PCs using the PCA loadings obtained for MIS 5a (Table S11), thus making them comparable. We calculated overlap values using the cloud of points produced by the union of both periods’ environmental conditions—points that did not fill the entire volume of the two ellipsoids but that represent a relevant background to take into account when performing the measurements, sensu^75^. The value of overlap, referred to as the Jaccard index (*J*)^76^, is the proportion of total points contained within the intersection of the two ellipsoids (A and B); *J* = A ⋂ B/A ⋃ B. To test for statistical significance, we compared the observed value of overlap against a distribution of overlap values calculated for 1000 pairs of ellipsoids created with data sampled randomly from each period’s background (background ellipsoids; sample size = number of archaeological occurrences per period). The null hypothesis for this test is that the ellipsoids fitted to actual observations overlap at least as much as ellipsoids created using the background samples (given that there is overlap). This hypothesis is rejected if the observed value is as extreme as, or more extreme than, the lower confidence limit (5%) of the distribution of overlap values derived from comparisons of background ellipsoids. We performed these analyses using two types of ellipsoids: (1) minimum volume ellipsoids (MVE), which adjust the centroid and covariance matrix to fit the defined proportion of points under consideration^77^; and (2) ellipsoids derived from a centroid and a covariance matrix (CVAE; common ellipsoids)^78^.

The R scripts and paleoclimatic variables used to conduct the analyses are available online at https://github.com/marlonecobos/Neanderthal.

## Supplementary Information

Supplementary Information 1.

Supplementary Information 2.
