# Supplementary material for: An ecological niche shift for Neanderthal populations in Western Europe 70,000 years ago
Source: Sci Rep. 2021 Mar 5;11:5346. doi: 10.1038/s41598-021-84805-6 (PMC7935894; doi:10.1038/s41598-021-84805-6)
Supplement: Supplementary file 1 — Supplementary Information 1. [file 41598_2021_84805_MOESM1_ESM.pdf]

Supplementary Information for

An ecological niche shift for Neanderthal populations in Western Europe 70,000 years ago.

William E. Banks*, Marie-Hélène Moncel, Jean-Paul Raynal, Marlon E. Cobos, Daniel Romero-Alvarez, Marie-Noëlle Woillez, Jean-Philippe Faivre, Brad Gravina, Francesco d'Errico, Jean-Luc Locht, Frédéric Santos

* Corresponding author: william.banks@u-bordeaux.fr

Supplementary Figures

Supplementary Tables

Supplementary References

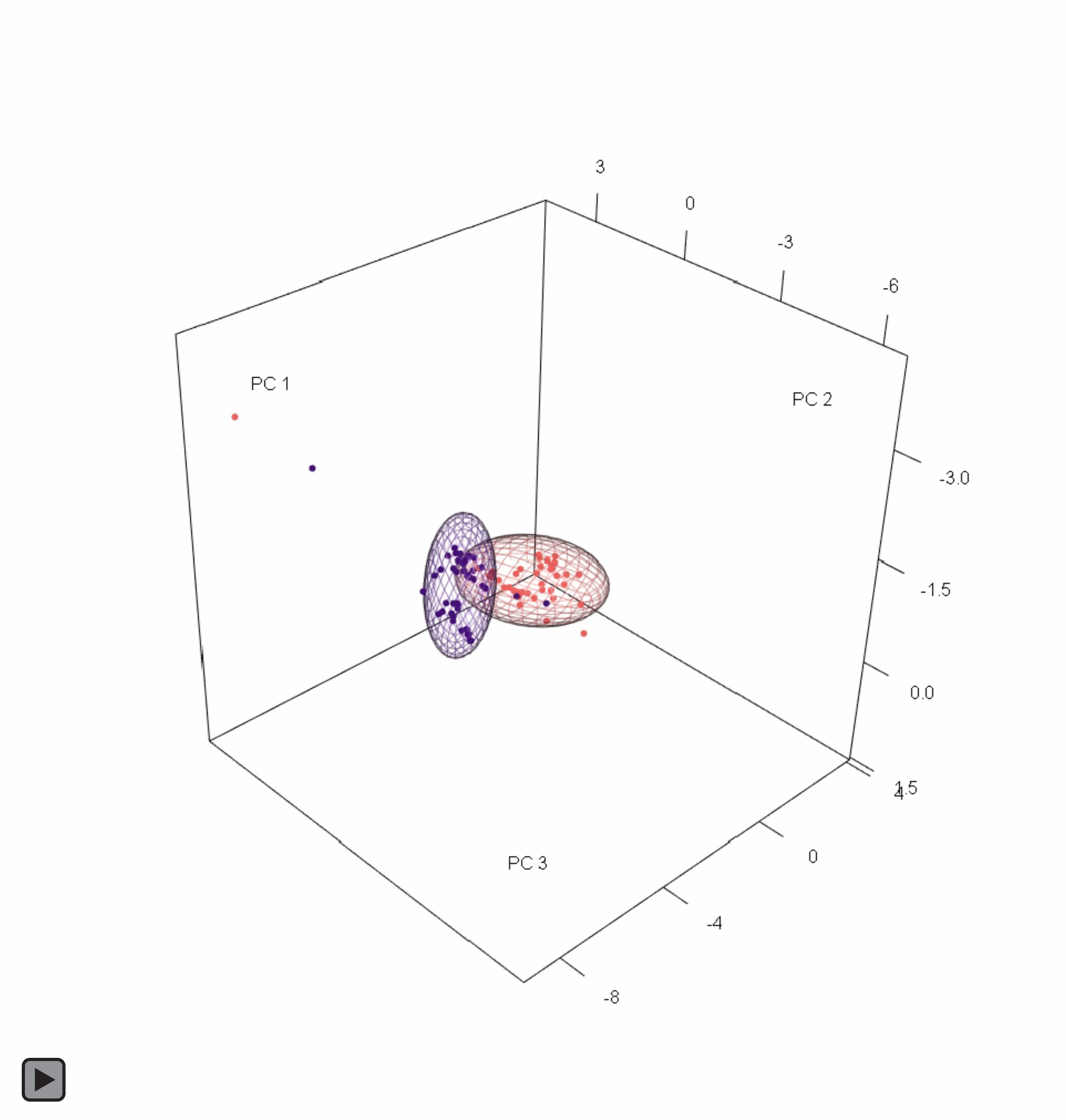

Figure S1. Animated presentation of Minimum volume ellipsoid (MVE) niche estimations for MIS 5a and MIS 4 Neanderthals.

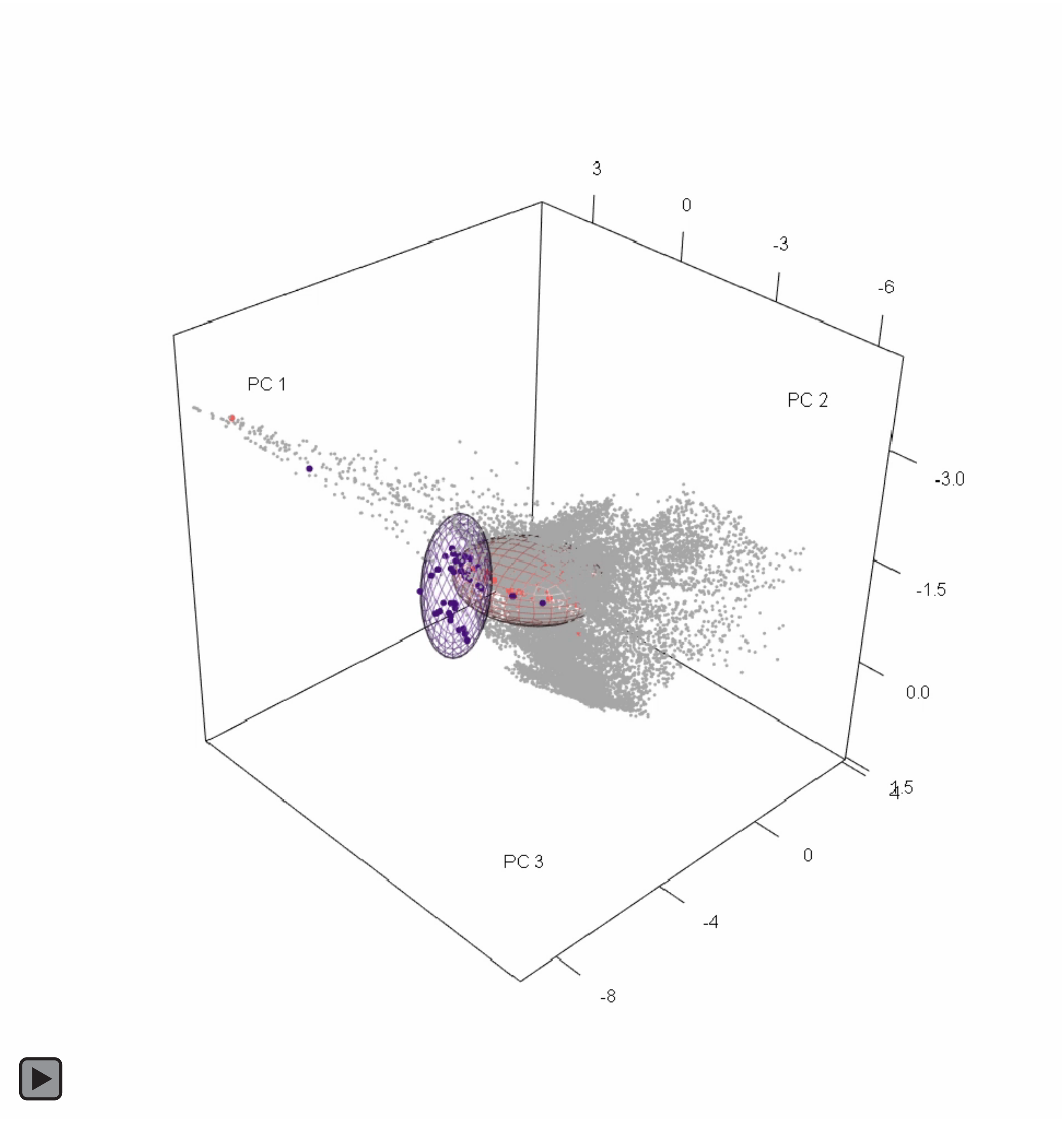

Figure S2. Animated presentation of Minimum volume ellipsoid (MVE) niche estimations for MIS 5a and MIS 4 Neanderthals against the MIS 5a environmental background.

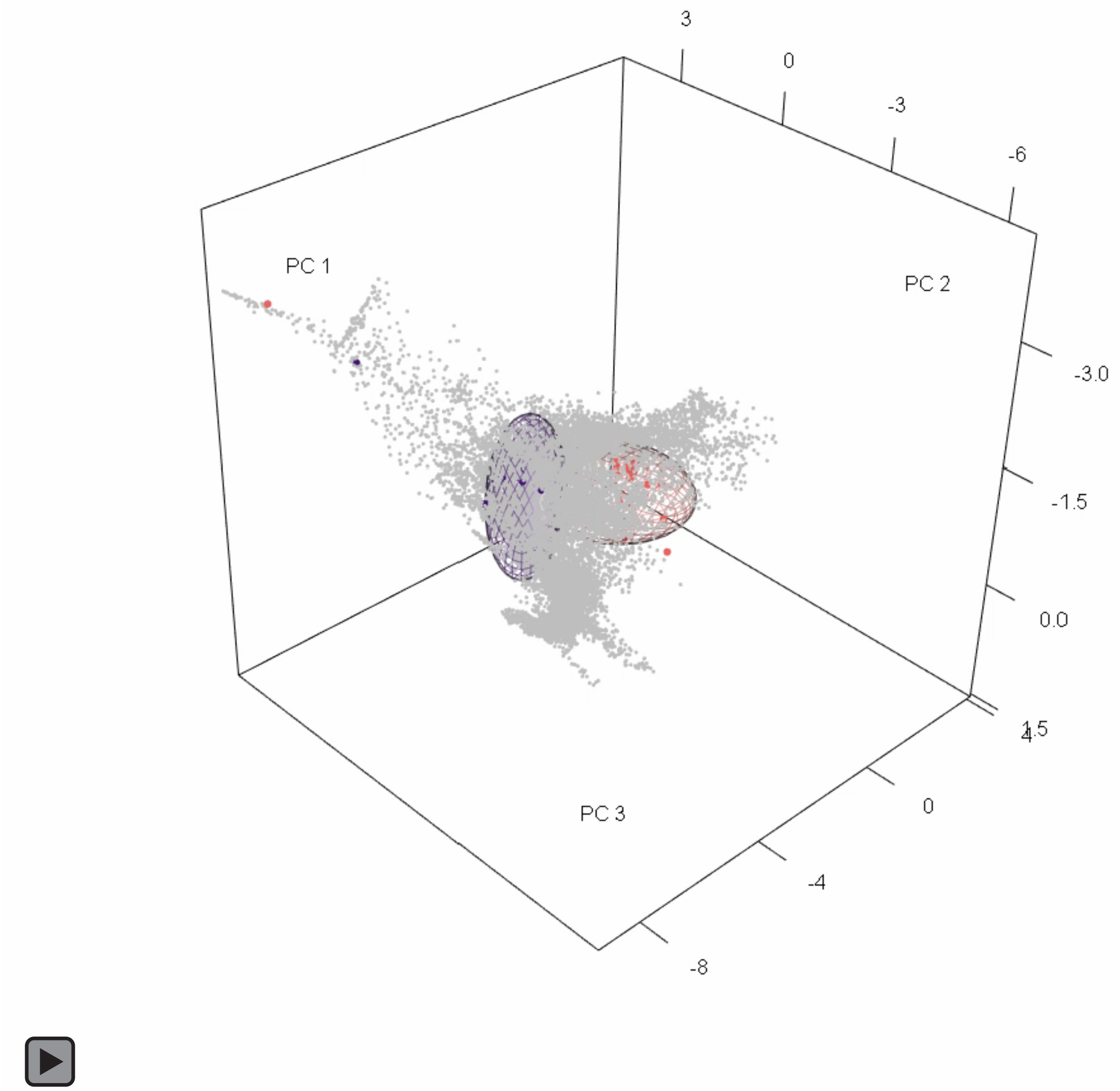

Figure S3. Animated presentation of Minimum volume ellipsoid (MVE) niche estimations for MIS 5a and MIS 4 Neanderthals against the MIS 4 environmental background.

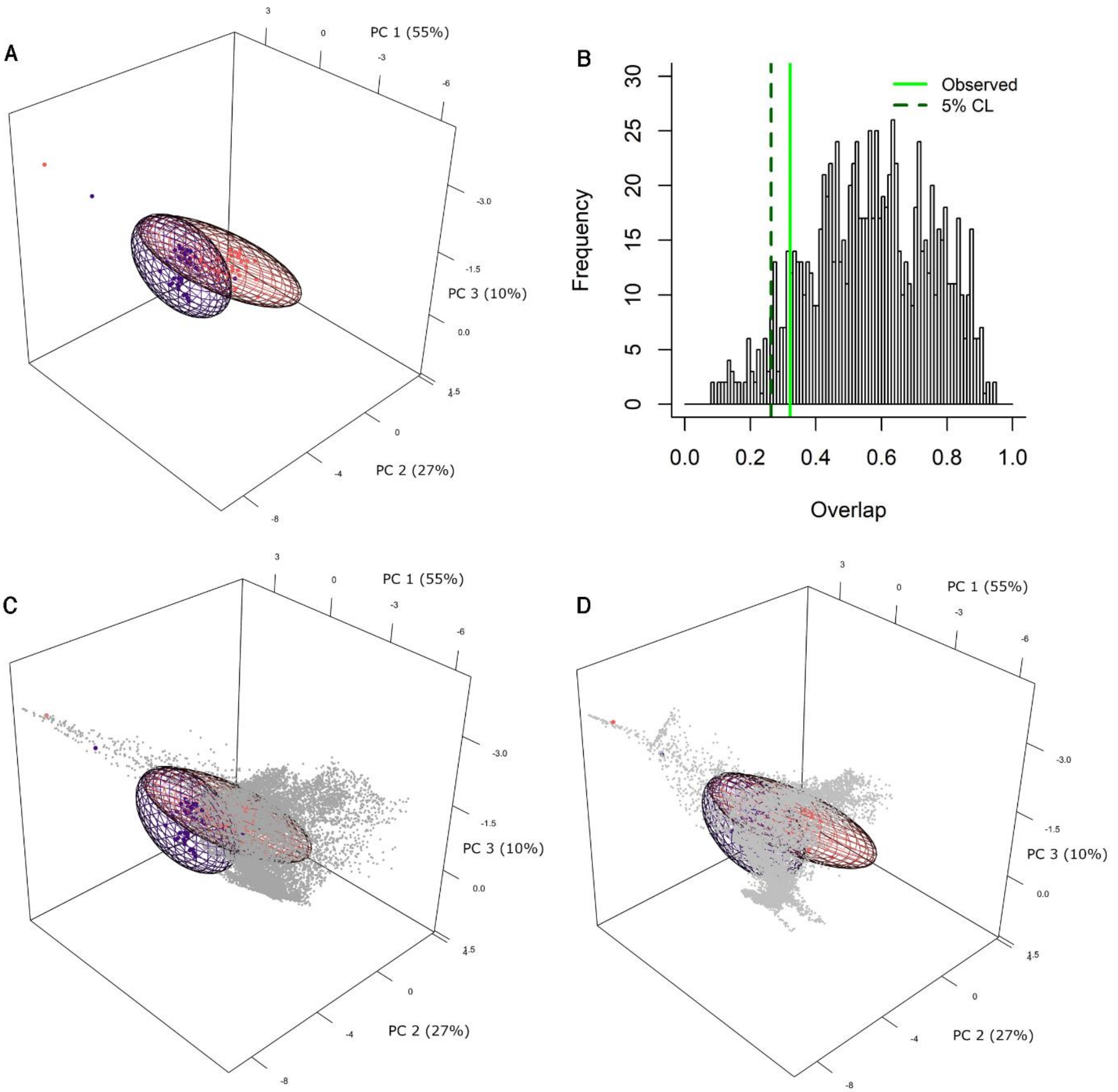

Figure S4. Centroid and covariance matrix ellipsoid (CVAE) niche estimations for MIS 5a and MIS 4 Neanderthals (A) and overlap comparison (B). MIS 5a occurrences and ellipsoid: red; MIS 5a environmental background: dark grey (C); MIS 4 occurrences and ellipsoid: blue; MIS 4 environmental background: light grey (D). Note that both MIS 5a and MIS 4 each have a single occurrence point (archaeological site) that falls outside the reconstructed ellipsoid. These two occurrence data fall below the error parameter *E* set at 5%.

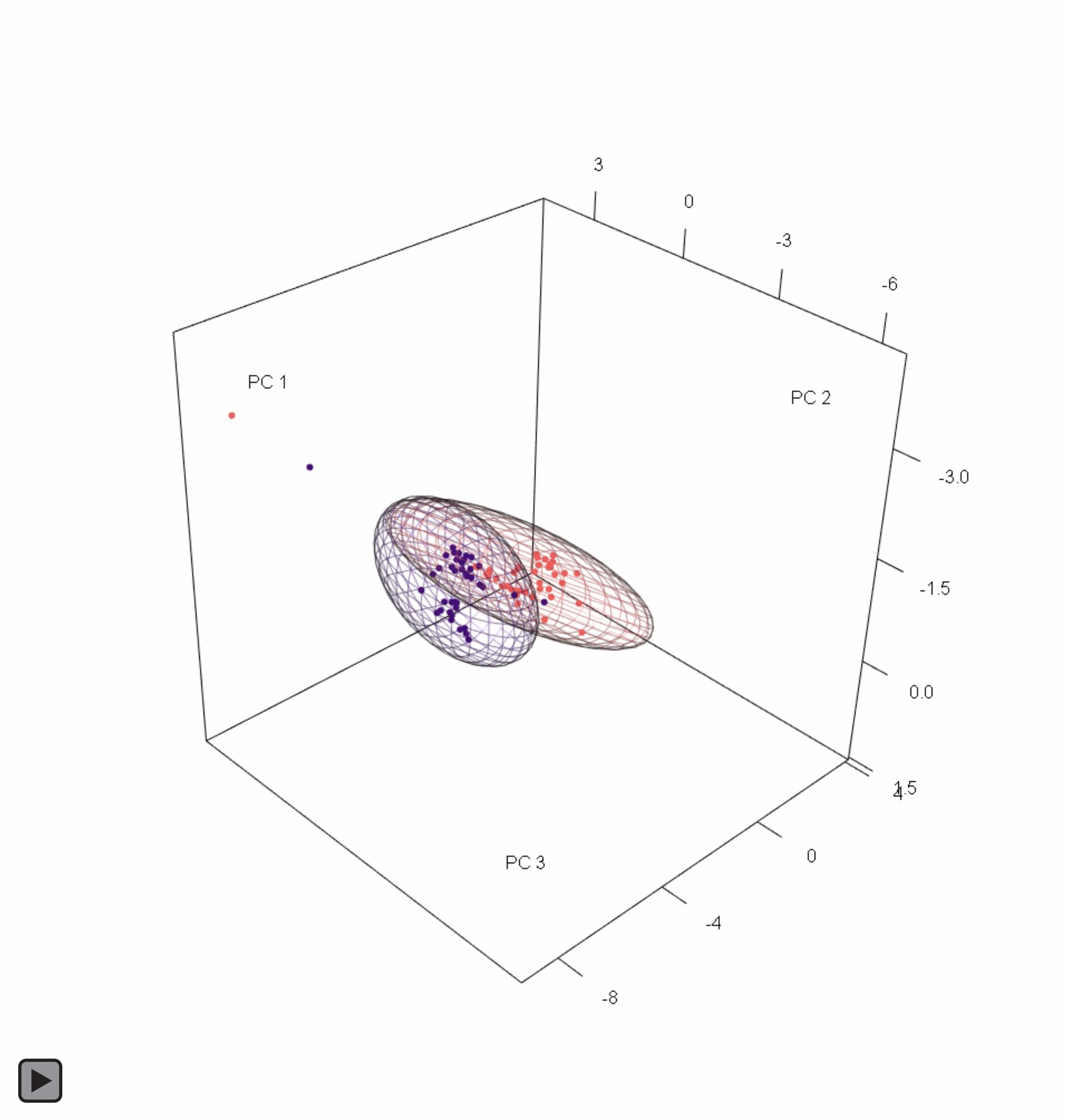

Figure S5. Animated presentation of centroid and covariance matrix ellipsoid (CVAE) niche estimations for MIS 5a and MIS 4 Neanderthals.

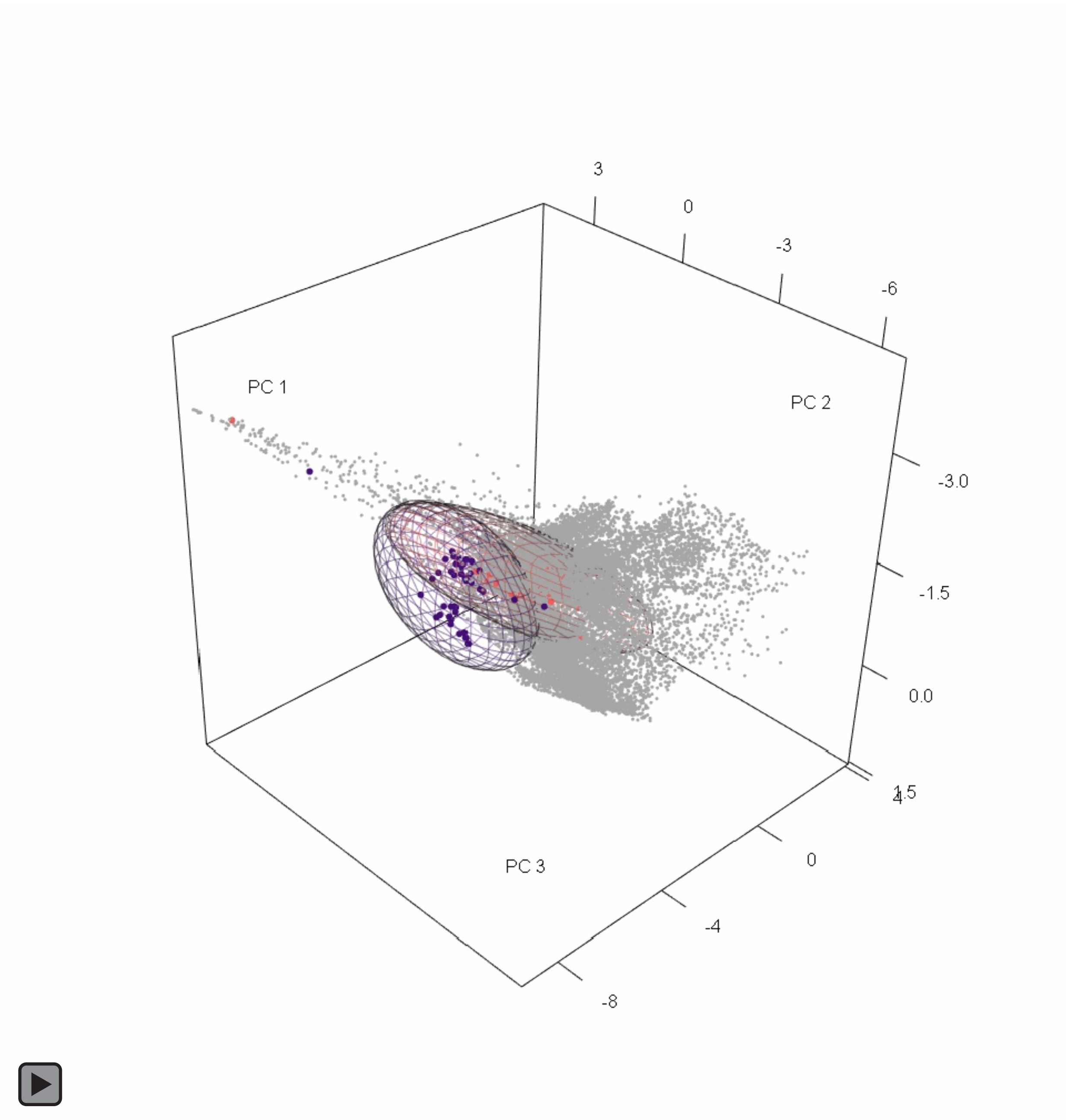

Figure S6. Animated presentation of centroid and covariance matrix ellipsoid (CVAE) niche estimations for MIS 5a and MIS 4 Neanderthals against the MIS 5a environmental background.

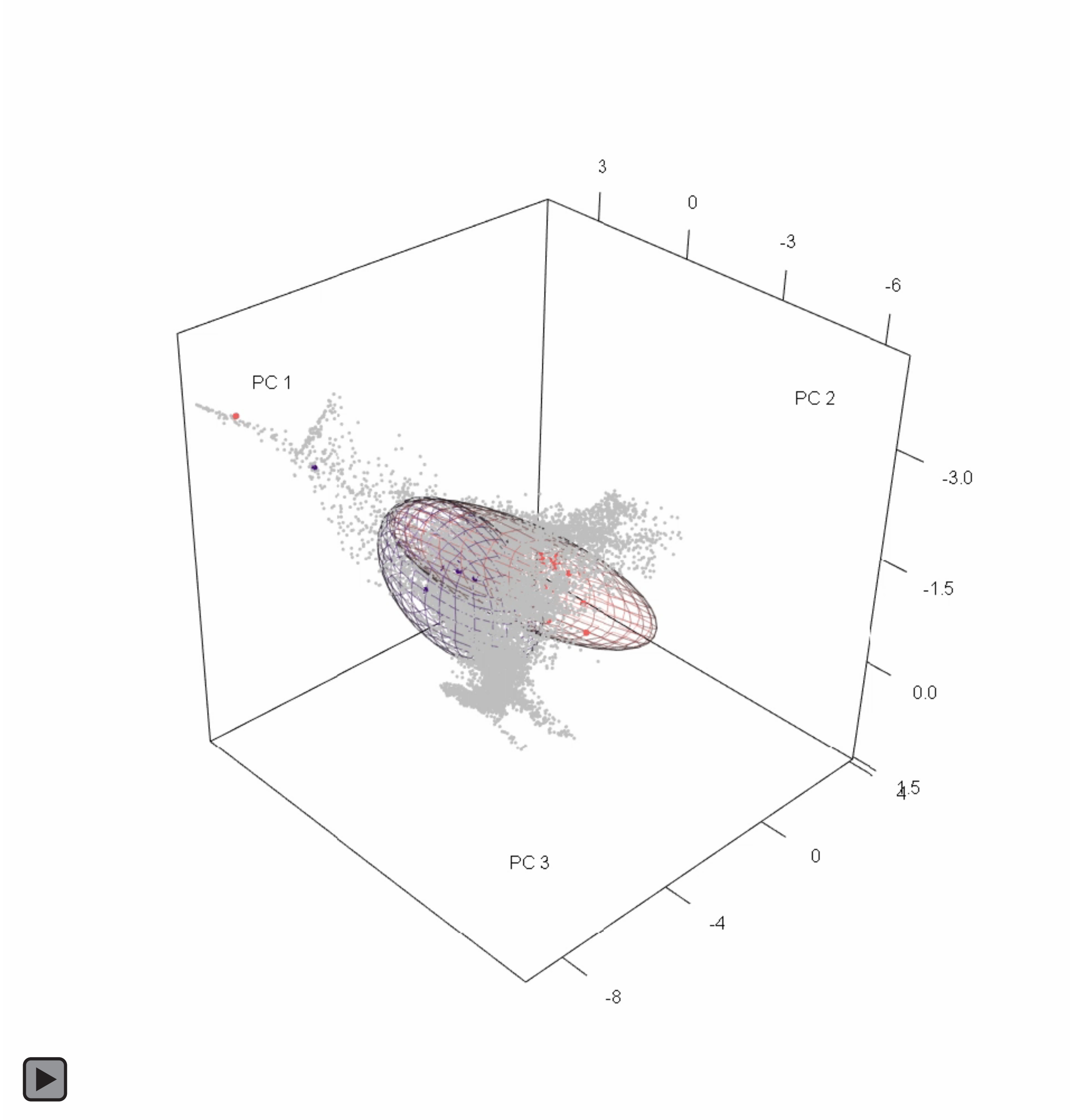

Figure S7. Animated presentation of centroid and covariance matrix ellipsoid (CVAE) niche estimations for MIS 5a and MIS 4 Neanderthals against the MIS 4 environmental background.

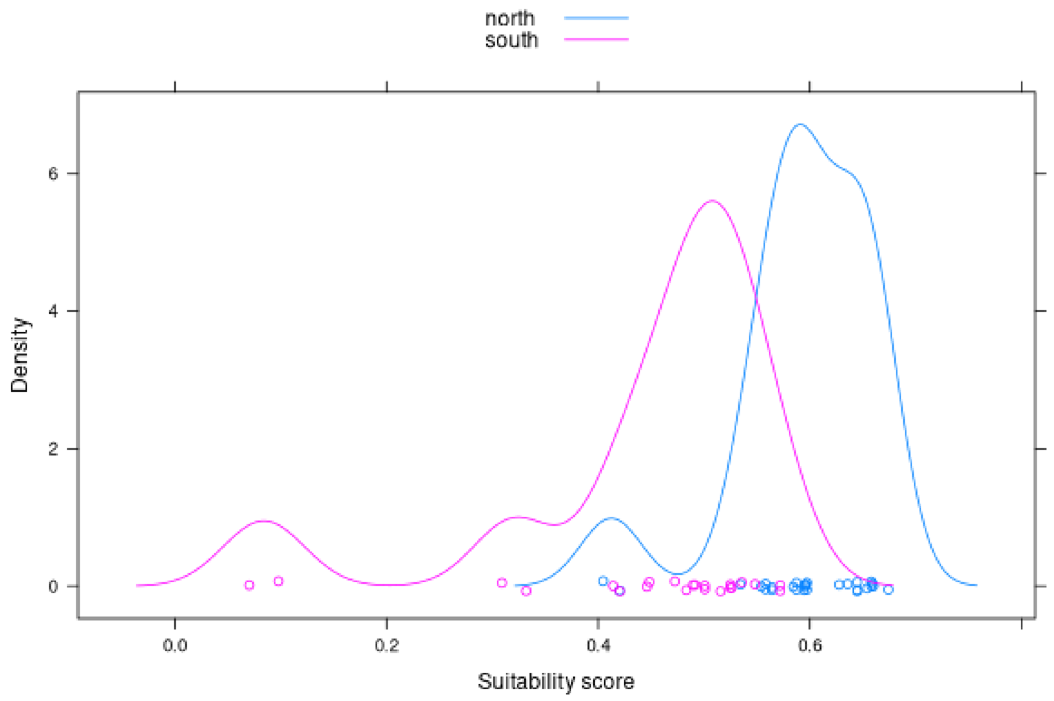

Figure S8. Density plot of suitability scores for the archaeological sites used to estimate the MIS 5a ecological niche.

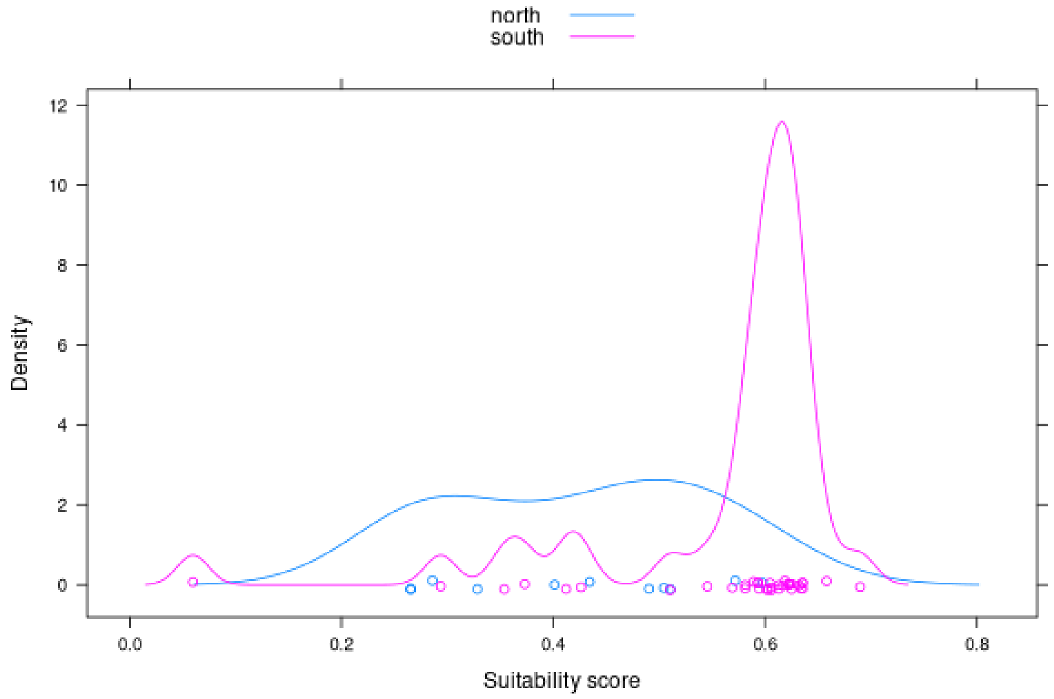

Figure S9. Density plot of suitability scores for the archaeological sites used to estimate the MIS 4 ecological niche.

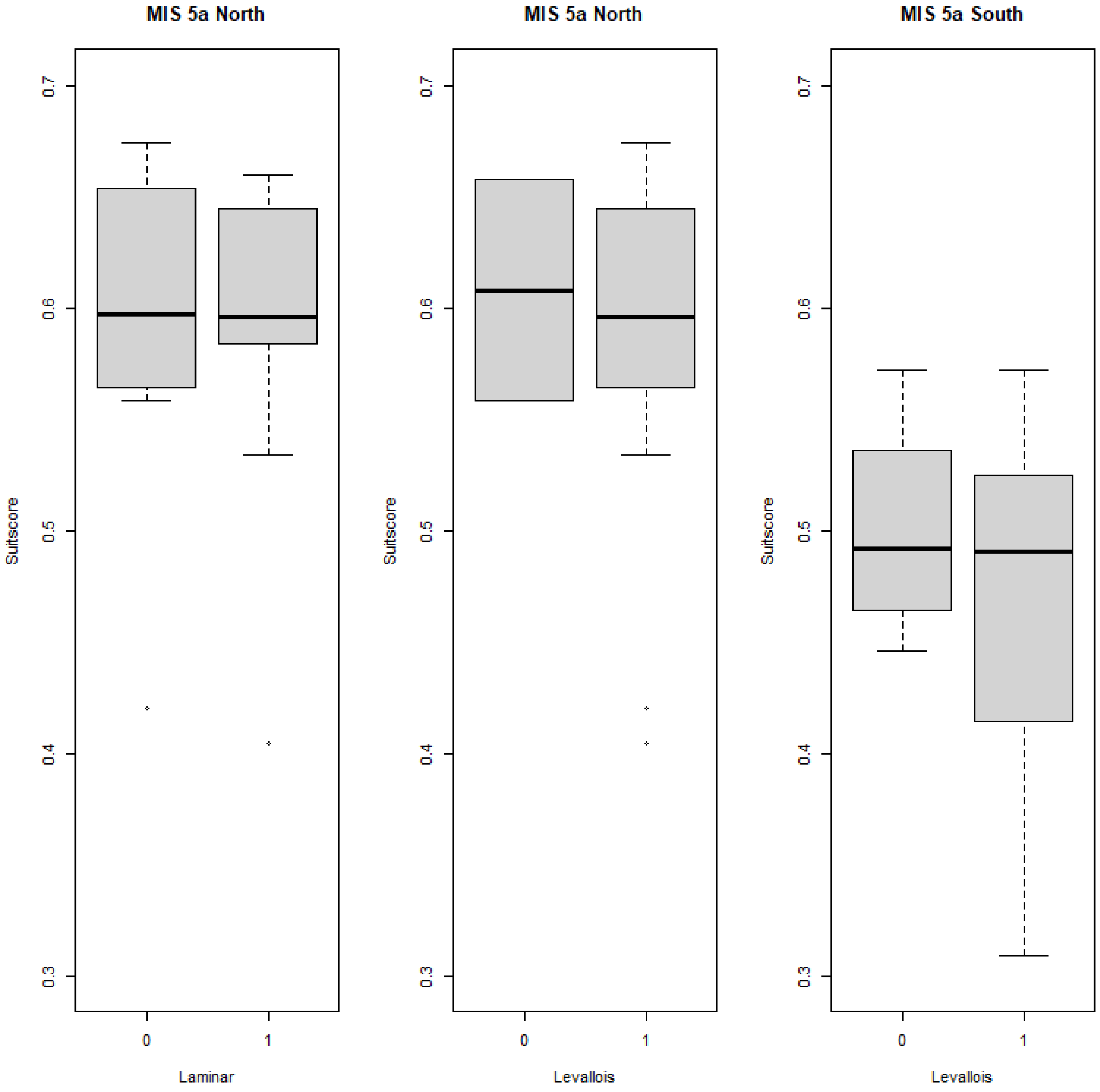

Figure S10. Boxplots of MIS 5a ecological niche estimation suitability scores (Suitscore) associated with the presence of lithic Technocomplexes (LTCs) that contain Laminar or Levallois production systems in the northern region of the study area and those with a Levallois component in the southern region. The 0 and 1 labels on the x-axis indicate the absence or presence of the lithic technology, respectively.

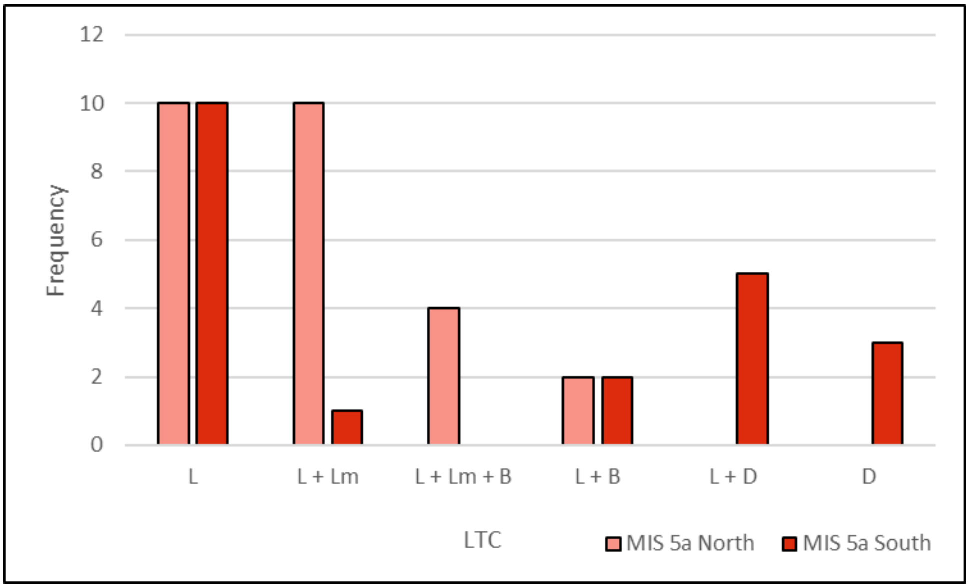

Figure S11. Lithic technocomplex (LTC) frequencies for the northern and southern portions of the study area during MIS 5a. See Tables S2 and S3 for LTC code explanations. A Fisher's Exact test demonstrates these differences to be significantly significant (***p*** **= 0.001**). LTCs absent in both study areas, as well as those coded as Indeterminate, are not depicted and were excluded from the Fisher's exact evaluation.

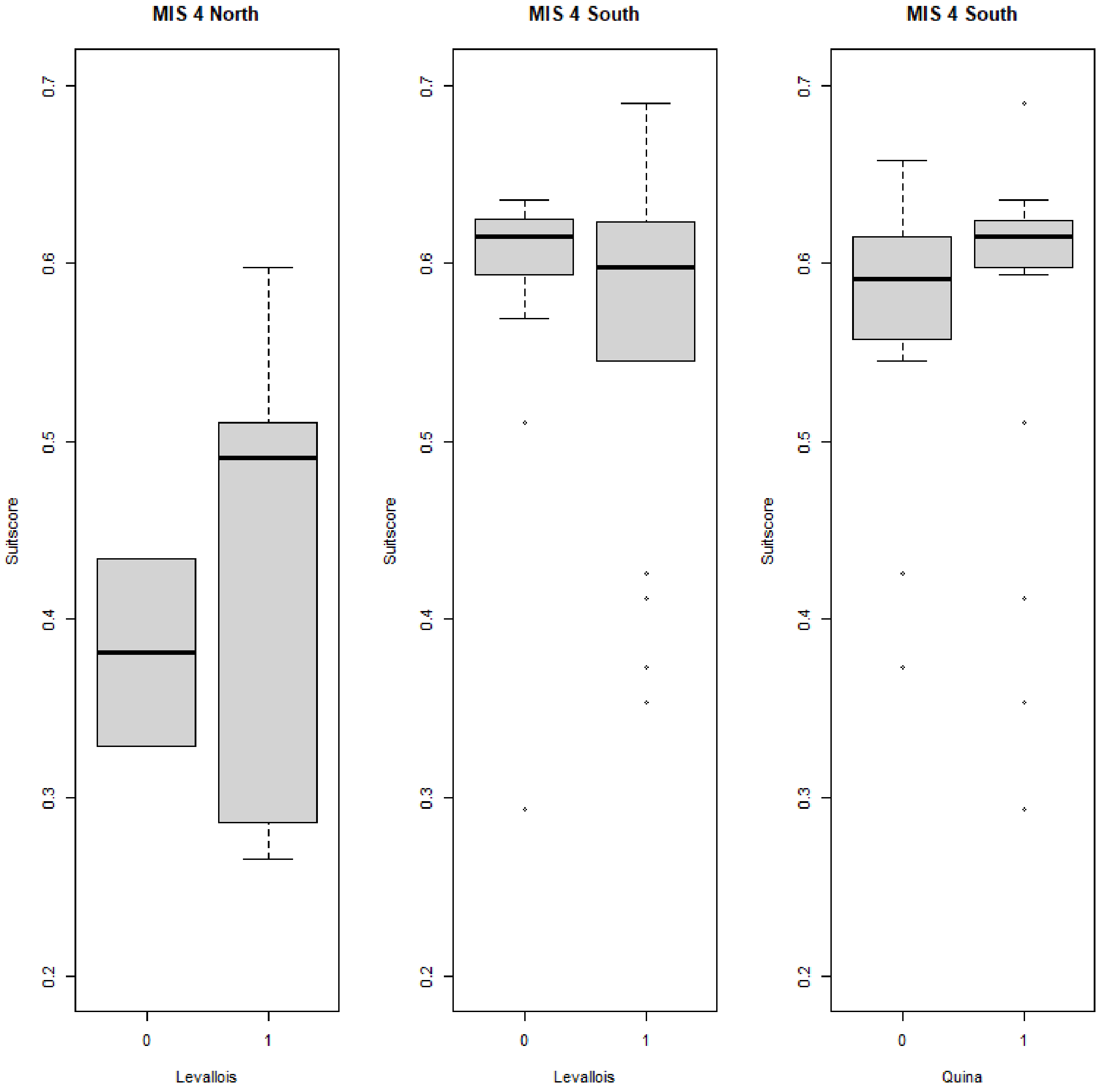

Figure S12. Boxplots of MIS 4 ecological niche estimation suitability scores (Suitscore) associated with the presence of lithic Technocomplexes (LTCs) that contain a Levallois component in the northern region of the study area and those that used Levallois or Quina production systems in the southern region. The 0 and 1 labels on the x-axis indicate the absence or presence of the lithic technology, respectively.

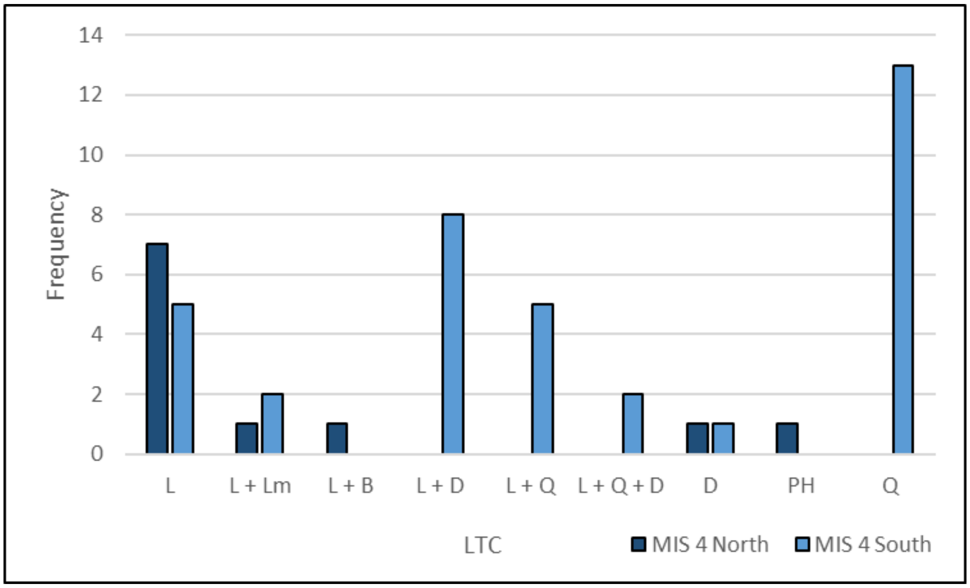

Figure S13. Lithic technocomplex (LTC) frequencies for the northern and southern portions of the study area during MIS 4. See Tables S2 and S3 for LTC code explanations. A Fisher's exact test demonstrates these differences to be significantly significant (***p* = 0.001**). LTCs absent in both study areas, as well as those coded as Indeterminate, are not depicted and were excluded from the Fisher's Exact evaluation.

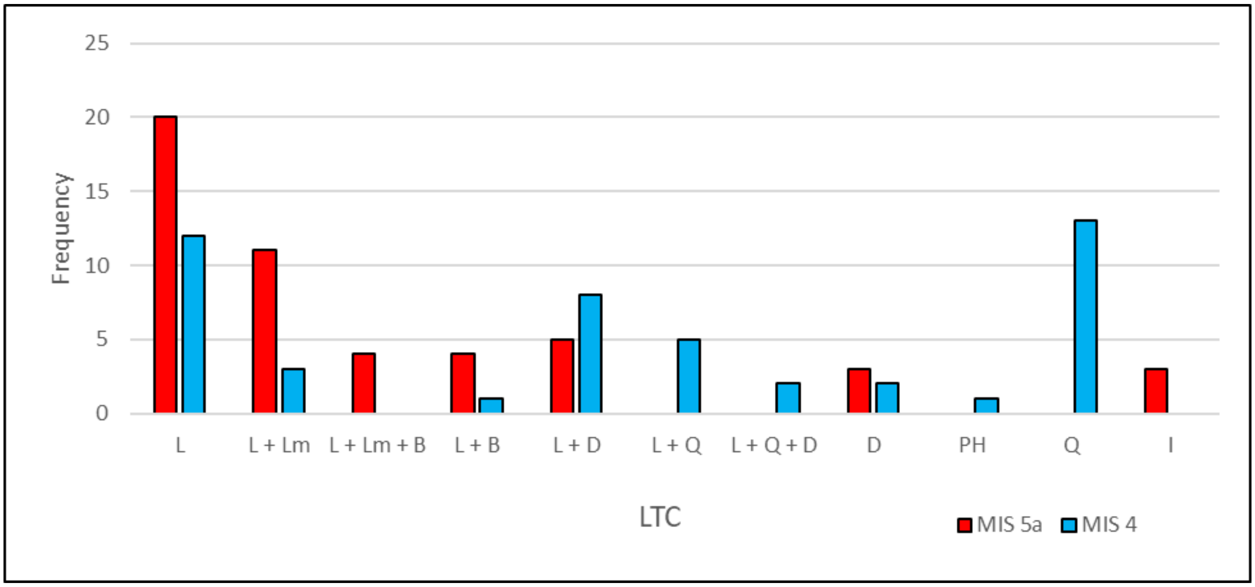

Figure S14. Lithic technocomplex (LTC) frequencies for the entire study area between MIS 5a and MIS 4. See Tables S2 and S3 for LTC code explanations. The presented frequencies are contained in Tables S2 and S3. A Fisher's exact test demonstrates these differences to be significantly significant (***p* = 0.001**).

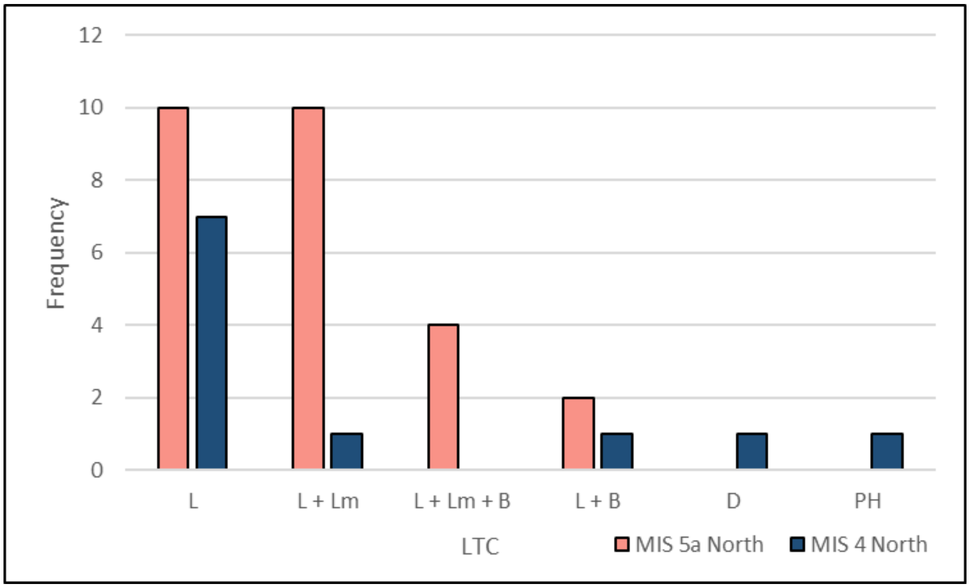

Figure S15. Lithic technocomplex (LTC) frequencies for MIS 5a and MIS 4 in the northern portion of the study area. See Tables S2 and S3 for LTC code explanations. A Fisher's exact test demonstrates these differences to be significantly significant (***p* = 0.049**). LTCs absent in the study area during these periods, as well as those coded as Indeterminate, are not depicted and were excluded from the Fisher's Exact evaluation.

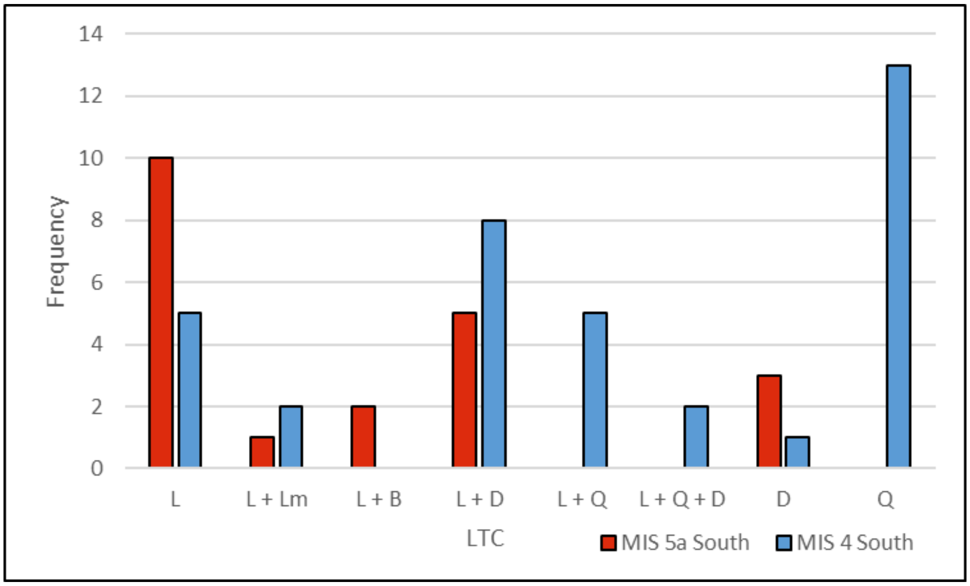

Figure S16. Lithic technocomplex (LTC) frequencies for MIS 5a and MIS 4 in the southern portion of the study area. See Tables S2 and S3 for LTC code explanations. A Fisher's exact test demonstrates these differences to be significantly significant (***p* = 0.001**). LTCs absent in the study area during these periods, as well as those coded as Indeterminate, are not depicted and were excluded from the Fisher's Exact evaluation.

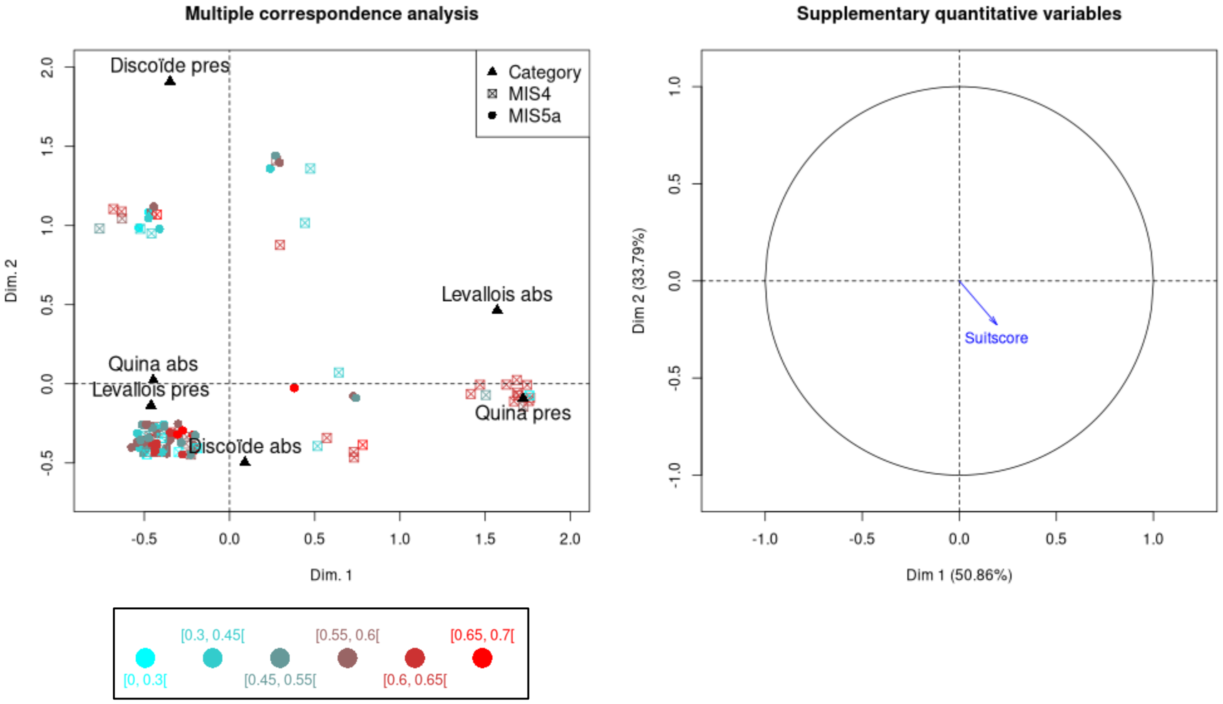

Figure S17. Results of a Multiple Correspondence Analysis performed on the presence (pres) and absence (abs) of Discoid, Levallois, and Quina lithic production systems observed in the archaeological assemblages from the sites used to estimate ecological niches for MIS 5a and MIS 4. In the left hand panel, suitability score intervals are color coded and attributed to each individual archaeological level. A jittering function was applied to each cluster of points in order to make all points visible since many site assemblages have the same axis coordinates. The right hand panel depicts suitability score (Suitscore) as a supplementary variable and its correlation to the two axes, although it did not actively participate in their construction.

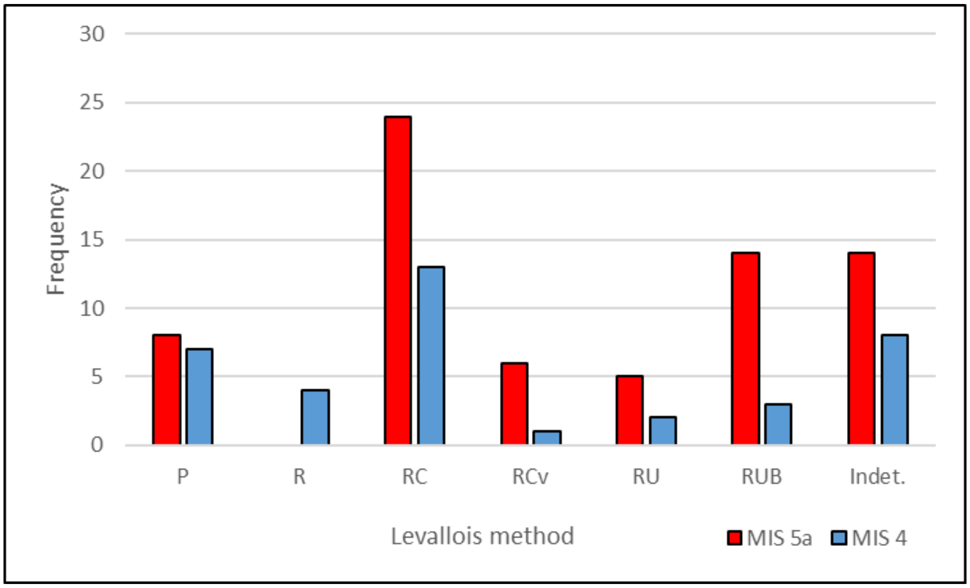

Figure S18. Levallois method frequencies for the entire study area between MIS 5a and MIS 4. See Tables S2 and S3 for Levallois method code explanations. A Fisher's exact test demonstrates that the differences between time periods are not significant (***p* = 0.07**).

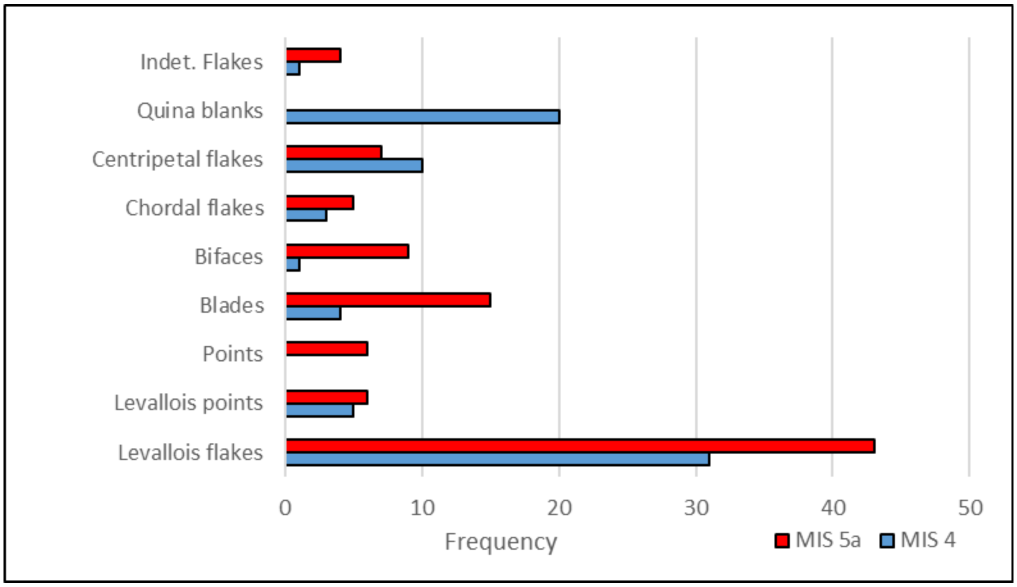

Figure S19. Frequencies of lithic technology products for the entire study area between MIS 5a and MIS 4. The presented frequencies are contained in Tables S2 and S3. A Fisher's exact test demonstrates these differences to be significantly significant (***p* = 0.001**).

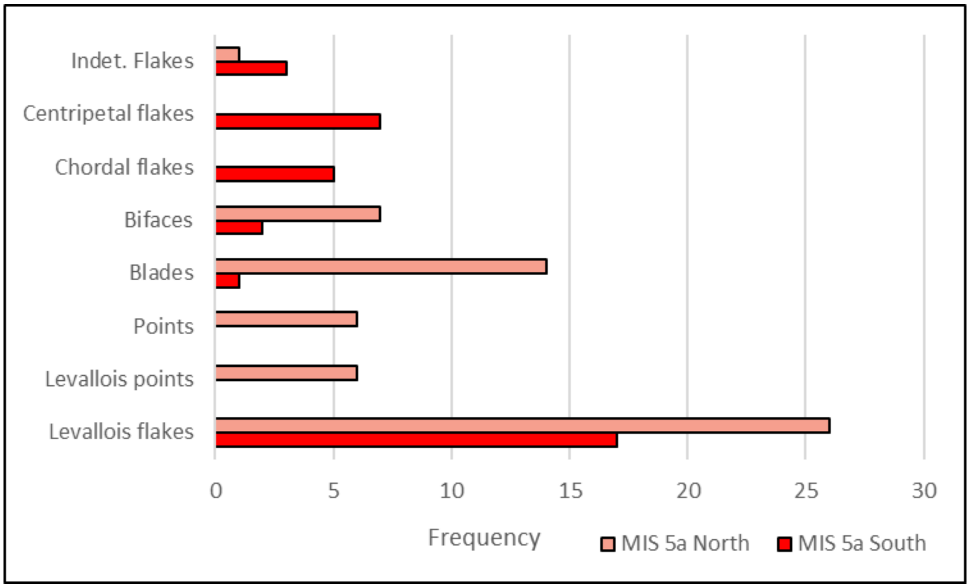

Figure S20. Frequencies of lithic technology products between the northern and southern portions of the study area during MIS 5a. These data are contained in Tables S2 and S3. A Fisher's exact test demonstrates these differences to be significantly significant (***p* = 0.001**). Products absent in both study areas are not depicted and were excluded from the Fisher's exact evaluation.

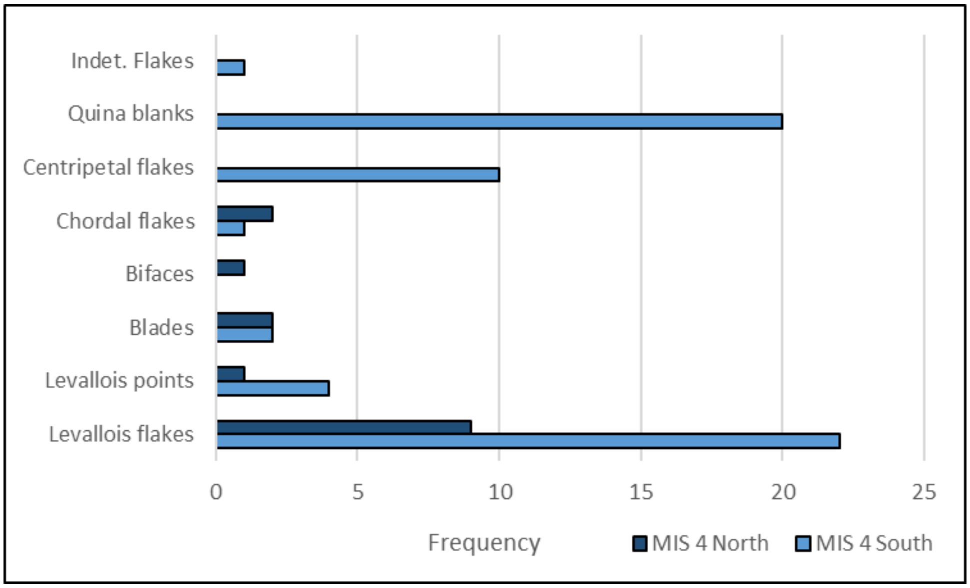

Figure S21. Frequencies of lithic technology products between the northern and southern portions of the study area during MIS 4. These data are contained in Tables S2 and S3. A Fisher's exact test demonstrates these differences to be significantly significant (***p* = 0.001**). Products absent in both study areas are not depicted and were excluded from the Fisher's exact evaluation.

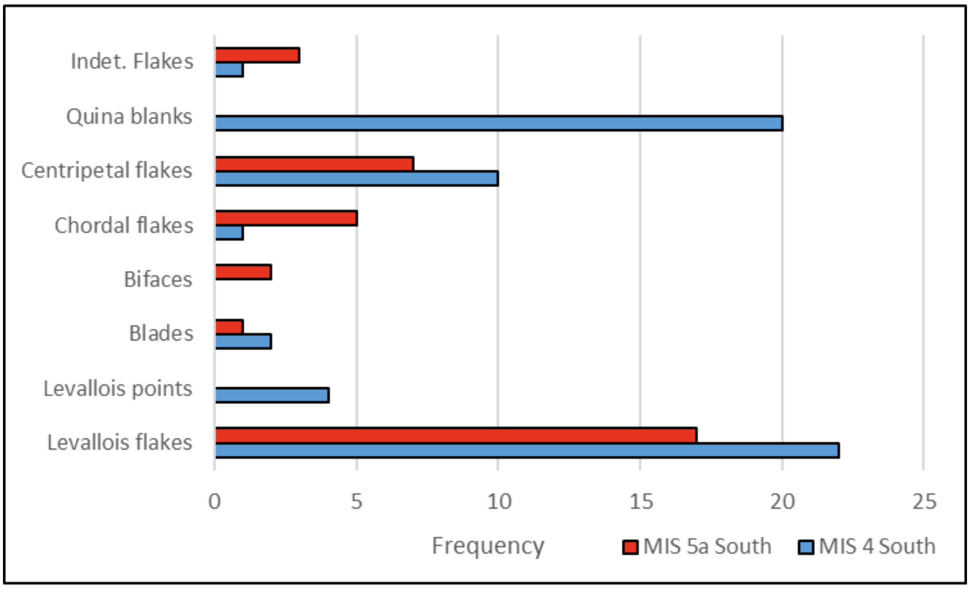

Figure S22. Frequencies of lithic technology products for MIS 5a and MIS 4 in the southern portion of the study area. These data are contained in Tables S2 and S3. A Fisher's exact test demonstrates these differences to be significantly significant (***p* = 0.001**). Products absent in both study areas are not depicted and were excluded from the Fisher's exact evaluation.

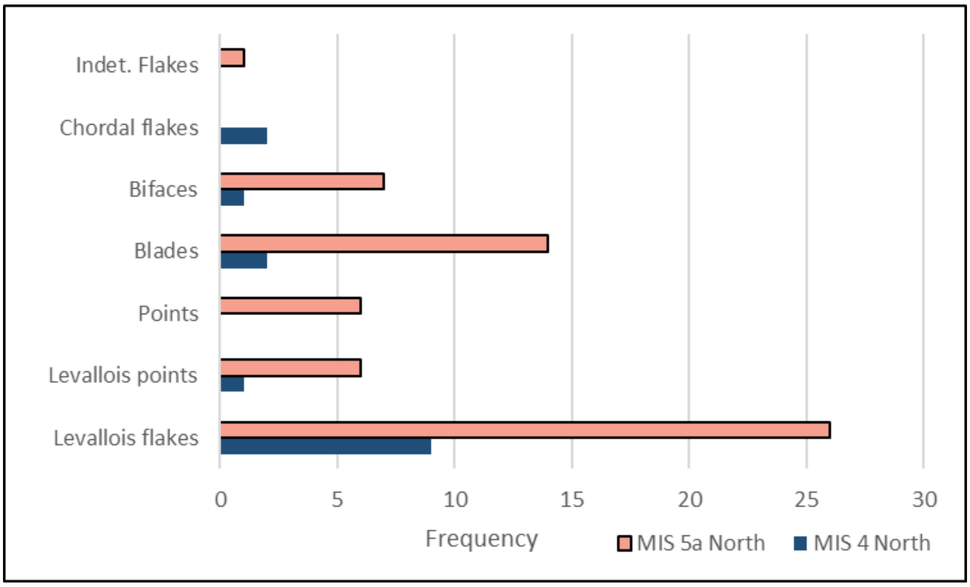

Figure S23. Frequencies of lithic technology products for MIS 5a and MIS 4 in the northern portion of the study area. These data are contained in Tables S2 and S3. A Fisher's exact test demonstrates that the differences between time periods in this portion of the study area are not significant (***p* = 0.15**). Products absent in both study areas are not depicted and were excluded from the Fisher's exact evaluation.

Table S1. Archaeological sites used to estimate late MIS 5 (5a) and MIS 4 Neanderthal niches. This table is an Excel spreadsheet contained in an individual tab in the Excel file available for download in "Supplementary Information."

Table S2. Techno-typological characteristics of MIS 5a archaeological contexts employed for estimating ecological niches. This table is an Excel spreadsheet contained in an individual tab in the Excel file available for download in "Supplementary Information."

Table S3. Techno-typological frequencies of the MIS 5a archaeological contexts employed for estimating ecological niches. This table is an Excel spreadsheet contained in an individual tab in the Excel file available for download in "Supplementary Information."

Table S4. Techno-typological characteristics of MIS 4 archaeological contexts employed for estimating ecological niches. This table is an Excel spreadsheet contained in an individual tab in the Excel file available for download in "Supplementary Information."

Table S5. Techno-typological frequencies of the MIS 4 archaeological contexts employed for estimating ecological niches. This table is an Excel spreadsheet contained in an individual tab in the Excel file available for download in "Supplementary Information."

Table S6. Minimum volume ellipsoid (MVE) estimations and comparison of Neanderthal niches during Marine Isotope Stage (MIS) 5a and MIS 4.

	**MIS 5a**	**MIS 4**					
Ellipsoid volume	5.804	2.018					
	**Total points**	**Overlapped points**	**Overlap**	**p value**	**Confidence Limit**	**Proportional size MIS5a vs MIS4**	**Proportional size MIS4 vs MIS5a**
MIS5a vs MIS4	11543	268	0.023	0.014	0.05	1.265	0.79

Table S7. Centroid and covariance matrix ellipsoid (CVAE) estimations and comparison of Neanderthal niches during Marine Isotope Stage (MIS) 5a and MIS 4.

	**MIS 5a**	**MIS 4**					
Ellipsoid volume	20.838	13.579					
	**Total points**	**Overlapped points**	**Overlap**	**p value**	**Confidence Limit**	**Proportional size MIS 5a vs MIS 4**	**Proportional size MIS 4 vs MIS 5a**
MIS 5a vs MI S4	18539	5959	0.321	0.103	0.05	1.206	0.829

Table S8. Archaeological site levels, Marine Isotope Stage (MIS) designations, lithic technocomplex (LTC) attributions, and associated ecological niche model suitability scores. This table is an Excel spreadsheet contained in an individual tab in the Excel file available for download in "Supplementary Information."

Table S9. Boundary conditions of the paleoclimatic simulations employed in this study.

**Simulation**	**Period**	**Ice sheets**	**CO2 (ppm)**	**CH4 (ppb)**	**N2O (ppb)**	**Obliquity**	**Precession Angle**	**Eccentricity**
**MIS 5a**	-80 ka	present-day	230	550	267	23.198	313.58	0.0288
**MIS 4**	-60 ka	ICE6-G - 16k	200	426	230	23.23	266.65	0.0184

Table S10. Principal Component Analysis summary.

	PC1	PC2	PC3	PC4	PC5
Standard deviation	1.6594	1.1649	0.7198	0.4648	0.3942
Proportion of variance	0.5507	0.2714	0.1036	0.0432	0.0311
Cumulative proportion	0.5507	0.8221	0.9257	0.9689	1.0000

Table S11. Principal Component Analysis loadings (rotations).

	PC1	PC2	PC3	PC4	PC5
biomass	-0.55815	0.133611	-0.05206	0.459257	-0.67601
ctemp	0.364381	0.54628	-0.5977	0.435213	0.148817
mprec	-0.49387	0.278789	-0.50361	-0.64888	0.060823
npp	-0.37843	0.556122	0.523017	0.167516	0.495893
wtemp	0.410586	0.544731	0.335961	-0.38802	-0.52082

References cited in Supplementary Tables S1, S2, and S4

1. Defleur, A. *et al.* Le niveau Moustérien de la grotte de l'Adaouste. *Bull. Mus. D'Anthropologie Préhistorique Monaco* **37**, 11–48 (1994).
2. Onoratini, G., Mafart, B., Joris, C. & Baroni, I. Les occupations humaines de la grotte de l'Adaouste (Jouques, Bouches-du-Rhône). *Quaternaire* **8**, 175–187 (1997).
3. Locht, J.-L. *et al.* Données inédites sur le Quaternaire et le Paléolithique du nord de la France. *Rev. Archéologique Picardie* **3**, 5–70 (2013).
4. Locht, J.-L. *et al.* Angé (loir-et-Cher): un site moustérien à influences multiples. in *Les plaines du Nord-Ouest: Carrefour de l'Europe au Paléolithique moyen?* (eds. Depaepe, P., Goval, É., Koehler, H. & Locht, J.-L.) 101–125 (Société Préhistorique Française, 2015).
5. Duran, J.-P. & Soler, N. Variabilité des modalités de débitage et des productions lithiques dans les industries moustériennes de la grotte de l'Arbreda, secteur alpha (Serinyà, Espagne). *Bull. Société Préhistorique Fr.* **103**, 241–262 (2006).
6. Soler, N. & Maroto, J. L'estratigrafia de la cova de l'Arbreda (Serinya, Girona). *Cypsela* **6**, 53–66 (1987).
7. Delagnes, A. *et al.* Le gisement Pléistocène moyen et supérieur d'Artenac (Saint-Mary, Charente) : premier bilan interdisciplinaire. *Bull. Société Préhistorique Fr.* **96**, 469–496 (1999).
8. Delagnes, A. & Tournepiche, J.-F. La Grotte d'Artenac (Saint-Mary, Charente). in *Préhistoire entre Vienne et Charente: Hommes et sociétés du Paléolithique* (eds. Buisson-Catil, J. & Primault, J.) 109–116 (Association des Publications Chauvinoises, 2010).
9. Goval, E., Hérisson, D., Locht, J.-L. & Coudenneau, A. Levallois points and triangular flakes during the Middle Palaeolithic in northwestern Europe: Considerations on the status of these pieces in the Neanderthal hunting toolkit in northern France. *Quat. Int.* **411**, 216–232 (2016).

10. Swinnen, C., Locht, J.-L. & Antoine, P. Le gisement moustérien d'Auteuil (Oise). *Bull. Société Préhistorique Fr.* **93**, 173–182 (1996).

11. Marchal, F. *et al.* Neandertals paleoenvironment in Western Provence: The contribution of Les Auzières 2 (Méthamis, Vaucluse, France). *Comptes Rendus Palevol* **8**, 493–502 (2009).

12. Daujeard, C. Balazuc - Grotte des Barasses II. *ADLFI Archéologie Fr. - Inf. Une Rev. Gall.* (2013).

13. Moncel, M.-H., Raynal, J.-P., Fernandes, P., Delvigne, V. & Hardy, B. Les différentes phases d'occupation de la cavité : fonction(s) du site. 4.1. Le matériel lithique. in *La Grotte des Barasses II (Balazuc) : entre Néandertaliens, bouquetins et carnivores. Des occupations du Pleistocène supérieur en moyenne vallée de l'Ardèche* (ed. Daujeard, C.) 109–128 (L'Association de liaison pour le patrimoine et l'archéologie en Rhône-Alpes et en Auvergne, 2019).

14. Boeda, E. *Barbas, Creysse*. 22 (1994).

15. de Bayle des Hermens, R. & Laborde, A. Le gisement moustérien de la Baume-Vallée (Haute-Loire). *Bull. Société Préhistorique Fr. Études Trav.* **62**, 512–527 (1965).

16. Raynal, J.-P. *et al.* Land-Use Strategies, Related Tool-Kits and Social Organization of Lower and Middle Palaeolithic Groups in the South-East of the Massif Central, France. *Quartär* **60**, 29–59 (2013).

17. Raynal, J.-P. *et al.* Neanderthal land-use and related tool-kits at the MIS 5/4 boundary in the South-East portion of the French Massif Central. in *Unravelling the Palaeolithic. Ten years of research at the Centre for the Archaeology of Human Origins (CAHO, University of Southampton)* (eds. Ruebens, K., Romanowska, I. & Bynoe, R.) 53–72 (Archaeopress, 2012).

18. Raynal, J.-P. *et al.* Paléolithique moyen dans le Sud du Massif central : les données du Velay (Haute-Loire, France). in *Données récentes sur les modalités de peuplement et sur le cadre chronostratigraphique, géologique et paléogéographique des industries du Paléolithique ancien et*

moyen en Europe (eds. Molines, N., Moncel, M.-H. & Monnier, J.-L.) 173–201 (John and Erica Hedges Ltd., 2005).

19. Raynal, J.-P. & Decroix, C. L'Abri de Baume-Vallée (Haute-Loire, France), site moustérien de moyenne montagne dans son contexte régional. *Arqueol. Porto* 17–42 (1987).
20. Raynal, J.-P. & Huxtable, J. Premières datations par thermoluminescence du Moustérien charentien du Velay (Massif central, France). *Comptes Rendus Académie Sci. Paris* **309**, 157–162 (1989).
21. Vaissié, E. *et al.* Techno-économie et signification culturelle de l'occupation moustérienne supérieure de Baume-Vallée (Haute-Loire). *Comptes Rendus Palevol* **16**, 804–819 (2017).
22. Locht, J.-L. *et al.* Timescales, space and culture during the Middle Palaeolithic in northwestern France. *Quat. Int.* **411**, 129–148 (2016).
23. Locht, J.-L. & Swinnen, C. Le débitage discoïde du gisement de Beauvais (Oise) : aspects de la chaîne opératoire au travers de quelques remontages. *Paléo* **6**, 89–104 (1994).
24. Richter, D. *et al.* Thermoluminescence dating of heated flint from the Mousterian site of Bérigoule, Murs, Vaucluse, France. *J. Archaeol. Sci.* **34**, 532–539 (2007).
25. Texier, J.-P. & Francisco-Ortego, I. Main technological and typological characteristics of the lithic assemblage from Level 1 at Bérigoule, Murs-Vaucluse, France. in *The Definition and Interpretation of Levallois Technology* (eds. Dibble, H. L. & Bar-Yosef, O.) vol. 23 213–226 (Prehistory Press, 1995).
26. *Bettencourt-Saint-Ouen (Somme): Cinq occupations paléolithiques au début de la dernière glaciation*. (Foundation de la Maison des sciences de l'Homme, 2002).
27. Depaepe, P., GUERLIN, O., Swinnen, C. & Antoine, P. Occupations du Paléolithique moyen à Blangy-Tronville (Somme). *Rev. Archéologique Picardie* **3**, 3–21 (1999).
28. Meignen, L. L'abri moustérien du Brugas à Vallabrix (Gard). *Gall. Préhistoire* **24**, 239–253 (1981).

29. Valladas, H., Mercier, N., Falguères, C. & Bahain, J.-J. Contribution des méthodes nucléaires à la chronologie des cultures paléolithiques entre 300 000 et 35 000 ans BP. *Gall. Préhistoire* **41**, 153–166 (1999).

30. Jaubert, J. Les archéo-séquences du Paléolithique moyen du Sud-Ouest de la France: quel bilan un quart de siècle après François Bordes. *CTHS* **29**, 235–253 (2012).

31. Meignen, L. *L'abri des Canalettes: un habitat moustérien sur les grands Causses (Nant, Aveyron). Fouilles 1980–1986*. (CNRS Editions, 1993).

32. Bourguignon, L. *et al.* L'occupation moustérienne de la Doline de Cantalouette II (Creysse, Dordogne) : spécificités technologiques et économiques, premiers résultats d'une analyse intégrée. in *Les sociétés du Paléolithique dans un grand Sud-Ouest de la France : nouveaux gisements, nouveaux résultats, nouvelles méthodes* (eds. Jaubert, J., Bordes, J.-G. & Ortega, I.) 133–150 (Société préhistorique française, 2008).

33. Bourguignon, L. *et al.* Les occupations paléolithiques découvertes sur la section Nord de la déviation de Bergerac: résultats préliminaires obtenus à l'issue des diagnostiques. *Préhistoire Sud-Ouest* **11**, 155–172 (2004).

34. Blaser, F., Bourguignon, L., Sellami, F. & Rios, J. Une série lithique à composante Laminaire dans le Paléolithique moyen du Sud-Ouest de la France : le site de Cantalouette 4 (Creysse, Dordogne, France). *Bull. Société Préhistorique Fr.* **109**, 5–33 (2012).

35. Combier, J. Lyon. *Gall. Préhistoire* **8**, 103–127 (1965).

36. Combier, J. Lyon. *Gall. Préhistoire* **5**, 229–306 (1962).

37. Locht, J.-L. Chronologie, espaces et cultures: le Paléolithique moyen de France septentrionale. (Aix-Marseille Université, 2018).

38. Sellier-Segard, N. Bilan des recherches sur le Paléolithique moyen de l'Aisne (Picardie) : étude des assemblages lithiques du Début Glaciaire weichselien au Pléniglaciaire moyen du Weichselien. in *Les*

Plaines du Nord-Ouest : Carrefour de l'Europe au Paléolithique moyen ? (eds. Depaepe, P., Goval, É., Koehler, H. & Locht, J.-L.) 75–99 (Société Préhistorique Française, 2015).

39. Bordes, F. La stratigraphie de la Grotte de Combe-Grenal, commune de Domme (Dordogne). Note préliminaire. *Bull. Société Préhistorique Fr.* **52**, 426–429 (1955).

40. Faivre, J.-Ph., Gravina, B., Bourguignon, L., Discamps, E. & Turq, A. Late Middle Palaeolithic lithic technocomplexes (MIS 5–3) in the northeastern Aquitaine Basin: Advances and challenges. *Quat. Int.* **433**, 116–131 (2017).

41. Sellier, N. & Coutard, S. Données récentes sur le Paléolithique moyen de l'Aisne : une occupation du Weichselien ancien à Courmelles. *Rev. Archéologique Picardie* **3**, 5–16 (2007).

42. Cliquet, D. Les occupations paléolithiques en Normandie dans leur contexte chronostratigraphique : « bribes archéologiques ». *Quaternaire* 303–314 (2013) doi:10.4000/quaternaire.6755.

43. Delagnes, A. & Ropars, A. *Paléolithique moyen en pays de Caux (Haute-Normadie)*. (1996).

44. Hérisson, D. & Goval, E. Du Paléolithique inferieur au début du Paléolithique supérieur dans le Nord de la France: lumière sur les premières découvertes du Canal Seine-Nord Europe. *Notae Praehistoricae* **33**, 91–104 (2013).

45. Coutard, S. & Cliquet, D. Chronostratigraphie des formations pléistocènes et peuplement paléolithique en contexte littoral : le Val de Saire (Normandie). *Bull. Société Préhistorique Fr.* **102**, 477–499 (2005).

46. Goval, É. & Locht, J.-L. Remontages, systèmes techniques et répartitions spatiales dans l'analyse du site weichselien ancien de Fresnoy-au-Val (Somme, France). *Bull. Société Préhistorique Fr.* **106**, 653–678 (2009).

47. Pautrat, Y. Le Moustérien de 'Genay' (Cote-d'Or). *Bull. Société Préhistorique Fr.* **82**, 138–142 (1985).

48. Antoine, P. *et al.* Paléoenvironnements pléistocènes et peuplements paléolithiques dans le bassin de la Somme (nord de la France). *Bull. Société Préhistorique Fr.* **100**, 5–28 (2003).

49. Antoine, P. *et al.* Les séquences loessiques pléistocène supérieur d'Havrincourt (Pas-de-Calais, France) : stratigraphie, paléoenvironnements, géochronologie et occupations paléolithiques. *Quat. Rev. Assoc. Fr. Pour Létude Quat.* 321–368 (2014) doi:10.4000/quaternaire.7278.

50. *Les chasseurs des steppes durant le dernier glaciaire en France septentrionale. Paléoenvironnement, techno-économie, approche fonctionnelle et spatiale du gisement d'Havrincourt*. (Presses Universitaires de Liège, 2018).

51. Vallin, L. & Masson, B. Behaviour towards lithic production during the Middle Palaeolithic: examples from Hermies le Champ Bruquette and Hermies le Tio Marche (Pas-de-Calais, France). in *Lithics in action. Papers from the conference Lithic studies in the year 2000* (eds. Walker, E. A., Wenban-Smith, F. & Healy, F.) 5–25 (Oxbow Books, 2004).

52. Vallin, L. Le site moustérien d'Houppeville (Seine-Maritime) : remontages et étude technologique. *Rev. Archéologique Picardie* **1**, 163–174 (1988).

53. Meignen, L. Le site moustérien charentien de Ioton (Beaucaire-Gard). Etude sédimentologique et archéologique. *Bull. Assoc. Fr. Pour Létude Quat.* **13**, 3–17 (1976).

54. Jaubert, J., Hublin, J.-J., McPherron, S. P. & Soressi, M. Le gisement paléolithique de Chez Pinaud à Jonzac (Charente-Maritime). in *Préhistoire entre Vienne et Charente: Hommes et sociétés du Paléolithique* (eds. Buisson-Catil, J. & Primault, J.) 117–121 (Association des Publications Chauvinoises, 2010).

55. Deloze, V., Depaepe, P., Gouedo, J.-M., Krier, V. & Locht, J.-L. *Le Paléolithique moyen dans le nord du Sénonais*. (Éditions de la Maison des Sciences de lHomme, 1994).

56. Depaepe, P. Pour une poignée de bifaces: les industries pauvres en bifaces du Paléolithique moyen de la vallée de la Vanne (Yonne–France). in *Les industries à outils bifaciaux du Paléolithique moyen d'Europe occidentale, Actes de la table-ronde internationale de Caen (octobre 1999), Liège, ERAUL* (ed. Cliquet, D.) vol. 98 135–140 (Université de Liège, 2001).

57. Chauchat, C. La station prehistorique de plein air de Lestaulan, quartier de Maignon, a Bayonne (Pyrenees Atlantiques). *Munibe* **46**, 3–22 (1994).

58. Baldeon, A. El yacimiento de Lezetxiki (Gipuzkoa, Pais Vasco). Los niveles musterienses. *Munibe* **45**, 3–97 (1993).

59. Cauche, D. Les cultures moustériennes en Ligurie italienne : analyse du matériel lithique de trois sites en grotte. *L'Anthropologie* **111**, 254–289 (2007).

60. Lecervoisier, B. Étude stratigraphique, sédimentologique, micromorphologique et paléoclimatique de remplissages de grottes du Pléistocène supérieur ancien de l'Europe méditerranéenne : Sites moustériens du Boquete de Zafarraya (Andalousie), de Madonna dell'Arma (Ligurie) et de Kalamakia (Laconie, Péloponnèse). (Muséum National d'Histoire Naturelle, 2003).

61. Moncel, M.-H. *et al.* Nouvelles données sur les occupations humaines du début du Pléistocène supérieur de la moyenne vallée du Rhône (France). Les sites de l'Abri des Pêcheurs, de la Baume Flandin, de l'Abri du Maras et de la Grotte du Figuier (Ardèche). *Quaternaire* **21**, 385–411 (2010).

62. Moncel, M.-H., Gaillard, C. & Patou-Mathis, M. L'abri du Maras (Ardèche) : une nouvelle campagne de fouilles dans un site Paléolithique moyen (1993). *Bull. Société Préhistorique Fr.* **91**, 363–368 (1994).

63. Locht, J.-L., Seller, N., Antoine, P., Koehler, H. & Debenham, N. Mauquenchy (Seine-Maritime, France) : mise en évidence de deux niveaux d'occupation paléolithique dans un sol gris forestier daté du SIM 5A (début glaciaire weichselien). *Quat. Rev. Assoc. Fr. Pour Létude Quat.* 247–257 (2013) doi:10.4000/quaternaire.6657.

64. Locht, J.-L. & Depaepe, P. Le Paléolithique moyen récent en France septentrionale. in *Les plaines du Nord-Ouest: Carrefour de l'Europe au Paléolithique moyen?* (eds. Depaepe, P., Goval, É., Koehler, H. & Locht, J.-L.) 61–74 (Société Préhistorique française, 2015).

65. Teheux, E. Observations préliminaires sur le site paleolithique moyen de la Minette à Fitz-James (Oise). *Archéopages* **1**, 30–37 (2000).

66. Locht, J.-L., Deloze, V., Pihuit, P. & Teheux, E. Molinons/Le Grand Chanteloup. in *Le Paléolithique moyen dans le nord du Sénonais (Yonne) : contexte géomorphologique, industries lithiques et chronostratigraphies* (eds. Deloze, V., Depaepe, P., Gouedo, J.-M., Krier, V. & Locht, J.-L.) 119–138 (Editions de la Maison des Sciences de l'Homme, 1994).

67. Cliquet, D. & Aubry, B. Apport du site Paléolithique moyen de Mont-Saint-Aignan / La Vatine (Seine-Martime) à la connaissance des processus de mise en œuvre des matières premières lithiques. *Haute-Normandie Archéologique* **11**, 37–48 (2006).

68. Defleur, A., Crégut-Bonnoure, É., Desclaux, E. & Thinon, M. Présentation paléo-environnementale du remplissage de la Baume Moula-Guercy à Soyons (Ardèche) : implications paléoclimatiques et chronologiques. *L'Anthropologie* **105**, 369–408 (2001).

69. Duran, J.-P. & Abelanet, J. Un moustérien méditerranéen à bifaces: le gisement de Moutou-la-Joliette (Espira de l'Agly, Pyrénées-Orientales France). *Préhistoire Anthropol. Méditerranéennes* 7–27 (2004).

70. Deschamps, M. Le Vasconien : révision de sa signification à partir des industries lithiques d'Olha I et II, d'Isturitz et de Gatzarria. *PALEO Rev. Archéologie Préhistorique* 103–126 (2009).

71. Jacobs, Z. *et al.* The age of three Middle Palaeolithic sites: Single-grain optically stimulated luminescence chronologies for Pech de l'Azé I, II and IV in France. *J. Hum. Evol.* **95**, 80–103 (2016).

72. McPherron, S. P. & Dibble, H. L. The lithic assemblages of Pech de l'Azé IV (Dordogne, France). *Préhistoire Eur.* **15**, 9–43 (1999).

73. Moncel, M.-H., Brugal, J.-P., Prucca, A. & Lhomme, G. Mixed occupation during the Middle Palaeolithic: Case study of a small pit-cave-site of Les Pêcheurs (Ardèche, south-eastern France). *J. Anthropol. Archaeol.* **27**, 382–398 (2008).

74. Texier, P.-J. *et al.* L'abri Pié Lombard à Tourrettes-sur-Loup (Alpes-Maritimes): Anciennes Fouilles, Nouvelles Données. *Bull. Mus. Anthropol. Préhistorique Monaco* 19–49 (2011).

75. Kervazo, B., Turq, A. & Diot, M.-F. Le site Moustérien de plein air de la Plane, commune de Mazeyrolles, Dordogne : note préliminaire. *Bull. Société Préhistorique Fr.* **86**, 268–274 (1989).

76. Jaubert, J. & Bismuth, J. Le Paléolithique moyen des Pyrénées centrales : esquisse d'un schéma chronologique et économique dans la perspective d'une étude comparative avec les documents ibériques. in *Pyrénées préhistoriques. Arts et sociétés* (eds. Delporte, H. & Clottes, J.) 9–26 (Editions du Comité des Travaux historiques et scientifiques, 1996).

77. Costamagno, S., Liliane, M., Cédric, B., Bernard, V. & Bruno, M. Les Pradelles (Marillac-le-Franc, France): A mousterian reindeer hunting camp? *J. Anthropol. Archaeol.* **25**, 466–484 (2006).

78. Maureille, B. Marillac-le-Franc – Les Pradelles. *ADLFI Archéologie Fr. - Inf. Une Rev. Gall.* (2008).

79. Gerber, J. P. Le site des Ramandils une faune du début du würmien II en languedoc-méditerranéen. *Bull. Assoc. Fr. Pour Létude Quat.* **10**, 7–12 (1973).

80. Moles, V. & Boutié, P. Contribution à la reconnaissance d'une microproduction au Paléolithique moyen : les industries de la grotte des Ramandils (Port-La Nouvelle, Aude, France). *L'Anthropologie* **113**, 356–380 (2009).

81. Bonifay, M.-F. La grotte du Régourdou (Montignac, Dordogne) Stratigraphie et industrie lithique moustérienne. *Anthropol.* **68**, 49–64 (1964).

82. Delpech, F. L'environnement animal des Moustériens Quina du Périgord. *Paléo* **8**, 31–46 (1996).

83. Jaubert, J., Kervazo, B., Quinif, Y., Brugal, J.-P. & O'Yl, W. Le site paléolithique moyen de Rescoundudou (Aveyron, France). datations U/Th et interprétation chronostratigraphique. *Anthropol.* **96**, 103–112 (1992).

84. Ameloot-Van Der Heijden, N. L'industrie laminaire du niveau C.A. du gisement paléolithique moyen de Riencourt-lès-Bapaume (Pas-de-Calais). *Bull. Société Préhistorique Fr.* **90**, 324–327 (1993).

85. Tuffreau, A., Heijden, N. A.-V. D. & Ducrocq, T. La fouille de sauvetage du gisement paléolithique moyen de Riencourt-lès-Bapaume (Pas-de-Calais) : premiers résultats. *Bull. Société Préhistorique Fr.* **88**, 202–209 (1991).
86. Thiébaut, C. L'industrie lithique de la couche III du Roc de Marsal (Dordogne) : le problème de l'attribution d'une série lithique au Moustérien à denticulés. *PALEO Rev. Archéologie Préhistorique* 141–168 (2003).
87. Turq, A. Le Moustérien de type Quina du Roc de Marsal (Dordogne). *Bull. Société Préhistorique Fr.* **82**, 46–51 (1985).
88. Thiébaut, C. Le Moustérien à denticulés: Variabilité ou diversité techno-économique? (Université d'Aix-Marseille I – Université de Provence, 2005).
89. Monnier, J.-L., Huet, B. & Laforge, M. Application of sedimentological analysis to correlation of eroded layers under beaches with local and regional Pleistocene stratigraphy: A contribution to geological dating of Palaeolithic sites, northern coast of Brittany, France. *Quat. Int.* **231**, 78–94 (2011).
90. Delporte, H. Le gisement paléolithique de La Rochette (commune de Saint-Léon-sur-Vézère, Dordogne). *Gall. Préhistoire* **5**, 1–22 (1962).
91. Duran, J.-P. & Tavoso, A. Les industries moustériennes de la Rouquette (Puycelci, Tarn, France). *L'Anthropologie* **109**, 755–783 (2005).
92. Tuffreau, A. Le gisement paléolithique inférieur et moyen de St-Just-en-Chaussée (Oise). *Cah. Archéologiques Picardie* **4**, 9–29 (1977).
93. Moncel, M.-H. Les niveaux moustériens de la grotte de Saint-Marcel (Ardèche) [Fouilles René Gilles. Reconnaissance de niveaux à débitage discoïde dans la vallée du Rhône]. *Bull. Société Préhistorique Fr.* **95**, 141–170 (1998).

94. Koehler, H., Drwila, G., Duplessis, M. & Locht, J.-L. Représentativité et mobilité du débitage laminaire au Weichsélien ancien dans le Bassin parisien. *Bull. Société Préhistorique Fr.* **111**, 5–17 (2014).

95. Combier, J. Le gisement Paléolithique moyen de Vergisson II (France, Saône-et-Loire). *Archeo-Inter-Publica* **1**, 1–18 (2001).

96. Fabre, M. Environnement et subsistance au Pléistocène supérieur dans l'est de la France et au Luxembourg : étude ostéologique des gisements de la Baume de Gigny (Jura), Vergisson II (Saône-et-Loire) et Oetrange (Luxembourg). (Université de Provence - Aix-Marseille I, 2010).

97. Depaepe, P. *Le paléolithique moyen de la vallée de la Vanne (Yonne) : matières premières, industries lithiques et occupations humaines*. (Société Préhistorique Française, 2007).

98. Depaepe, P. Analyses spatiales par répartition proportionnelle des artefacts : premiers résultats sur deux sites du Paléolithique moyen (Lailly. *Bull. Société Préhistorique Fr.* **94**, 435–442 (1997).

99. Lebegue, F. & Meignen, L. Quina ou pas ? Révision techno-économique d'un site moustérien charentien en Languedoc oriental : la grotte de la Roquette à Conqueyrac (Gard, France). *Bull. Société Préhistorique Fr.* **111**, 603–630 (2014).
